# Adipocyte lipolysis drives acute stress-induced insulin resistance

**DOI:** 10.1038/s41598-020-75321-0

**Published:** 2020-10-23

**Authors:** Vidisha Raje, Katelyn W. Ahern, Brittany A. Martinez, Nancy L. Howell, Vici Oenarto, Mitchell E. Granade, Jae Woo Kim, Smanla Tundup, Katharina Bottermann, Axel Gödecke, Susanna R. Keller, Alexandra Kadl, Michelle L. Bland, Thurl E. Harris

**Affiliations:** 1 Department of Pharmacology, University of Virginia, Charlottesville, VA USA; 2 Department of Medicine, Endocrinology and Metabolism, University of Virginia, Charlottesville, VA USA; 3 Department of Medicine, Pulmonary and Critical Care Medicine, University of Virginia, Charlottesville, VA USA; 4 Institute of Cardiovascular Physiology, Heinrich Heine University Düsseldorf, Düsseldorf, Germany

**Keywords:** Fat metabolism, Homeostasis, Trauma

## Abstract

Stress hyperglycemia and insulin resistance are evolutionarily conserved metabolic adaptations to severe injury including major trauma, burns, or hemorrhagic shock (HS). In response to injury, the neuroendocrine system increases secretion of counterregulatory hormones that promote rapid mobilization of nutrient stores, impair insulin action, and ultimately cause hyperglycemia, a condition known to impair recovery from injury in the clinical setting. We investigated the contributions of adipocyte lipolysis to the metabolic response to acute stress. Both surgical injury with HS and counterregulatory hormone (epinephrine) infusion profoundly stimulated adipocyte lipolysis and simultaneously triggered insulin resistance and hyperglycemia. When lipolysis was inhibited, the stress-induced insulin resistance and hyperglycemia were largely abolished demonstrating an essential requirement for adipocyte lipolysis in promoting stress-induced insulin resistance. Interestingly, circulating non-esterified fatty acid levels did not increase with lipolysis or correlate with insulin resistance during acute stress. Instead, we show that impaired insulin sensitivity correlated with circulating levels of the adipokine resistin in a lipolysis-dependent manner. Our findings demonstrate the central importance of adipocyte lipolysis in the metabolic response to injury. This insight suggests new approaches to prevent insulin resistance and stress hyperglycemia in trauma and surgery patients and thereby improve outcomes.

## Introduction

Stress-induced hyperglycemia and insulin resistance after trauma and injury are well documented^1,2^. Post-injury abnormalities in glucose metabolism are observed in all patients, even without prior history of diabetes. While stress-induced mobilization of fuel is an evolutionary adaptation that was necessary to feed tissues in critical need, in the current clinical environment, stress hyperglycemia and insulin resistance are maladaptive. They are associated with adverse outcomes such as increased risk of infection, delayed wound healing, episodes of cardiovascular dysfunction, and increased mortality^3,4^. Insulin is currently used to control post-operative stress hyperglycemia. However, insulin therapy increases the risk of hypoglycemia and does not address the underlying insulin resistance^5,6^. Hence, understanding the early metabolic changes leading to insulin resistance and impaired glucose metabolism during severe trauma or surgical injury is key to finding better therapeutic approaches and improving outcomes.

Stress resulting from severe trauma, burns, or hemorrhagic shock (HS) triggers the release of counterregulatory hormones (e.g. catecholamines, glucagon, vasopressin, and glucocorticoids) that rapidly promote mobilization of stored energy and impair insulin action^2^. Glucagon, cortisol, and catecholamines stimulate hepatic glycogenolysis and gluconeogenesis, resulting in increased hepatic glucose output. Catecholamines also potently stimulate adipocyte triacylglycerol (TAG) breakdown (lipolysis) into glycerol and non-esterified fatty acids (NEFA) that further contribute to endogenous glucose production (EGP)^7–10^. While increased glucose levels after injury most likely result from increased hepatic glucose production, the cause of stress-induced impairments in peripheral glucose utilization is currently unknown.

Adipose tissue is an important energy reserve of stored TAGs for periods of critical need such as starvation or stress^11^. In addition, adipose tissue is an important endocrine organ regulating whole body glucose and lipid metabolism, and defects in adipocyte function affect whole body insulin sensitivity and glucose homeostasis^12,13^. Adipose tissue is among the earliest tissues to become insulin resistant in animal models of trauma^14,15^. We previously demonstrated that, in isolated adipocytes, impairments in insulin signaling after catecholamine exposure are reversed when lipolysis is inhibited^16^. In this study, we investigated the specific role of adipocyte lipolysis in regulating the early development of insulin resistance in vivo in response to acute stress resulting from surgical injury and HS. Our data suggests adipocyte lipolysis may be a possible therapeutic target for reducing the maladaptive acute stress response.

## Material and methods

### Animals

All animals were bred and maintained in accordance with the University of Virginia Animal Care and Use Committee regulations and the study was approved by the ACUC ethics committee. 10- to 12-week old male and female mice were used for studies unless otherwise indicated. *Atgl* (*Pnpla2*) knockout (B6;129P2-*Pnpla2*^*tm1Rze*^/J, Jax #019003), *Atgl*^*flox/flox*^ (B6N.129S-*Pnpla2*^*tm1Eek*^/J, Jax #024278), AdipoQ-cre (B6;FVB-Tg(Adipoq-cre)^*1Evdr/J*^, Jax #010803), and C57BL/6 J (Jax #000664) were from the Jackson Laboratory. *Aqp7*^−/−^ (*Aqp7*^*Gt(NAISTrap_TPM2–118)*Yais^)^17^ sperm was obtained from Riken Resource Center (Japan) and used to re-derive mice at the University of Virginia’s GEMM core. Adipocyte-specific ATGL knockout (FATA^−/−^) mice were generated by crossing *Atgl*^*flox/flox*^ with AdipoQ-cre mice, and control mice were age and sex-matched litter mates. *Atgl*^*flox/flox*^, cre- are described as WT^fl/fl^ and *Atgl*^*flox/flox*^, cre + as FATA^−/−^.

### Animal treatments

Atglistatin (Cayman Chemicals) and GS-9667 (Gilead Sciences) in DMSO were diluted in vehicle (1% Cremophore-EL in saline) and administered i.p. 4 h and 1 h respectively prior to start of the experiment. For vehicle treatments, animals received matched volume of DMSO in 1% Cremophor-EL. For clamp studies, vehicle or GS-9667 was administered i.p. 30 min prior to start of the experiment. For glycerol treatments, a bolus of glycerol (3 mg/g) was administered i.p. immediately before the start of the HS protocol.

### Hemorrhagic shock protocol

Surgical trauma and hemorrhage (HS) were induced as previously described^18^, with a few modifications. Briefly, 10- to 12-week old mice were fasted 4 h before experiments, then mice were anesthetized with isoflurane inhalation, shaved, restrained in a supine position, and kept anesthetized by continuous inhalation of 1.5% isoflurane and 98.5% air throughout the surgical procedure. A 2-cm ventral midline laparotomy was performed representing soft tissue trauma. The abdomen was then closed using 6-0 Ethilon sutures. A polyethylene-10 catheter was placed in the right femoral artery for monitoring of mean arterial pressure (MAP). Mice were bled to a MAP of 35–40 mm Hg within 10 min and maintained at this pressure for 30 min. At the end of 30 min, mice were injected with saline or insulin (Humulin R, Eli Lily) (0.5 U) into the inferior vena cava, and 4 min later tissues were harvested. Blood glucose and lactate levels were measured at the tail vein by a handheld glucometer (One-touch Ultra) and lactate monitor (Nova Biomedical), respectively.

### Hyperinsulinemic–euglycemic clamps

Hyperinsulinemic–euglycemic clamps were conducted as previously described^19^, with a few modifications. Mice were fasted 2–4 h prior, anesthetized with 2% isoflurane, and maintained at 1.5% isoflurane thereafter. An incision was made to expose and isolate the left jugular vein, and a catheter was placed for infusions. In the induction period (− 30 to 0 min), mice were infused with D-[3-^3^H] glucose at 0.17 μCi/min and either epinephrine (1 μg/kg/min) or saline. Following the induction period, the hyperinsulinemic–euglycemic clamp was started with a continuous infusion of insulin at 10 mU/kg/min and either epinephrine (1 μg/kg/min) or saline. Dextrose was infused at variable rates to maintain plasma glucose at 150 mg/dL throughout the clamp. Insulin-stimulated whole body glucose turnover rates were determined by a continuous infusion of D-[3-^3^H] glucose at 0.1 μCi/min throughout the clamp. Insulin-stimulated glucose uptake in peripheral tissues was estimated by administering a bolus injection of 13 μCi of [U-^14^C]-2-deoxy-d-glucose (PerkinElmer) at 120 min after the start of the clamp. Serum samples were collected from the tail vein at various time points throughout the clamp for measuring plasma [^3^H] glucose and [U-^14^C]-2-deoxy-d-glucose concentrations. Blood glucose was measured at the tail vein with a handheld glucometer (One Touch Ultra 2).

The concentration of D-[3-^3^H]-glucose in serum was measured according to the procedure of Vanderbilt-MMPC with some modifications. Briefly, 6 μl of plasma sample mixed with 14 μl saline was treated with 100 µl of 3 N Ba(OH)_2_ and ZnSO_4_, and 100 μl of supernatant was pipetted into a scintillation vial and dried in the oven overnight. Scintillation fluid was added to the dried vial or to 100 μl non-dry supernatant for measuring the radioactivity in a liquid scintillation counter. 2-[U-^14^C]-deoxy-d-glucose was measured in tissues as described previously^20^. Briefly, tissues were extracted and homogenized in 2 mL water. A fraction of the supernatant was used to determine total ^14^C radioactivity by passing through an ion exchange column (AG1-X8 beads, BioRad) to extract labeled 2-[U-^14^C]-deoxy-d-glucose phosphate. Radioactivity was then eluted and counted using a scintillation counter. This counted fraction was compared with another fraction of the supernatant to obtain unfiltered ^14^C counts. The difference between the two samples was used to calculate accumulated 2-[U-^14^C]-deoxy-d-glucose phosphate. Finally, 2-DG uptake was determined by dividing the determined accumulated 2-[U-^14^C]-deoxy-d-glucose phosphate by the plasma glucose-specific activity area under the curve.

### Measurement of serum metabolites, hormones, cytokines, and adipokines

Tissue triglyceride levels and serum glycerol concentration were measured by Serum Triglyceride Determination Kit (TR0100, Sigma). NEFA was measured by HR series NEFA-HR kit (Wako Diagnostics). Commercial ELISA kits were used according to the manufacturer’s instructions to detect catecholamines (BAE5400, Rocky Mountain Diagnostics), insulin and corticosterone (98880 and 80556, Crystal Chem Inc.), and resistin (ELM-Resistin-1, Raybiotech). The limit of detection (LOD) for the insulin measurements is set at 0.05 ng/mL. Samples below the LOD are not included for statistical calculations. All other circulating cytokines and adipokines in serum were detected by Luminex Multiplex Assays at the University of Virginia flow core facility.

### Glycogen measurement

Glycogen was measured using Amplex Red method. Briefly, liver samples were homogenized in PBS and incubated with or without 1 mg/mL amyloglucosidase (AMG) in 0.2 M NaOAc, pH 4.8 for 1 h at 37 °C to hydrolyze glycogen into glucose. Samples were incubated with 0.25 U/mL glucose oxidase (G7141, Sigma), 0.17 U/mL horseradish peroxidase (P8250, Sigma), and 20 µM Amplex Red (A36006, Invitrogen) in 1 mM EDTA, 1 mM MgCl_2_, 10 µM FAD (F6625, Sigma), and 100 mM KH_2_PO_4_, pH 6.8 for 10 min in the dark at room temperature. Amplex Red emission (587 nm) was measured following excitation at 535 nm. Free glucose was subtracted from total AMG-hydrolyzable glucose to give glycogen values.

### Western blot and quantitative analysis

Tissues were homogenized in lysis buffer (1 mM EDTA, 1 mM EGTA, 1 mM DTT, 0.1% Tween 20, 10 mM sodium phosphate, and 50 mM β-glycerophosphate, pH 7.4) supplemented with protease inhibitors. Homogenates were centrifuged at 16,000×*g* for 10 min. Protein concentrations were determined using BCA (Pierce). Protein samples were boiled in Laemmli buffer and equal amounts of proteins (30 µg) were separated electrophoretically by SDS-PAGE and transferred to PVDF. Western blotting was carried out with antibodies to β-tubulin (Sigma-Aldrich), β-actin, ATGL, Akt, and pAkt^S473^ (Cell Signaling Technology). Blots were imaged by detecting alkaline phosphatase conjugated chemiluminescence on a Fujifilm LAS-4000 imager. For quantitation, membranes were probed for pAkt^S473^and normalized to total Akt, by stripping and re-immunoblotting the same membrane or by immunoblotting a separate membrane when necessary. In the latter condition equal loading between pAkt and total Akt membranes was achieved by immunoblotting a separate loading control (e.g., β-tubulin or β-actin). Blots were quantified by densitometry using ImageJ software and normalization was carried out in GraphPad Prism software by setting the largest relative value in each immunoblot to 100 and all other values adjusted accordingly.

### RNA extraction and real time qPCR

Total RNA was isolated with Trizol Reagent (Invitrogen), and samples were treated with DNase (Promega). cDNA was synthesized from 2 μg RNA with the Tetro complementary DNA Synthesis Kit, and real-time qPCR was performed with the SensiMix SYBR and Fluorescein Kit (Bioline). All samples were assayed in duplicates and analyzed with a CFX96 Real-Time PCR Detection System (Bio-Rad). Data normalized to 18S rRNA.

### Statistical analysis

Data are presented as mean ± SEM. Statistical analyses were performed using two-way ANOVA, except immunoblot quantitation which contained three independent variables was analyzed by three-way ANOVA. For all ANOVA analyses, all meaningful comparisons (differing by one variable) were performed and adjusted for. All statistically significant comparisons are denoted in the figures. The numbers for each group are reported in the figure legend. GraphPad Prism software was used for statistical analysis.

## Results

### Genetic inhibition of lipolysis during acute adrenergic stress prevents development of hyperglycemia and peripheral insulin resistance

**Figure 1:**
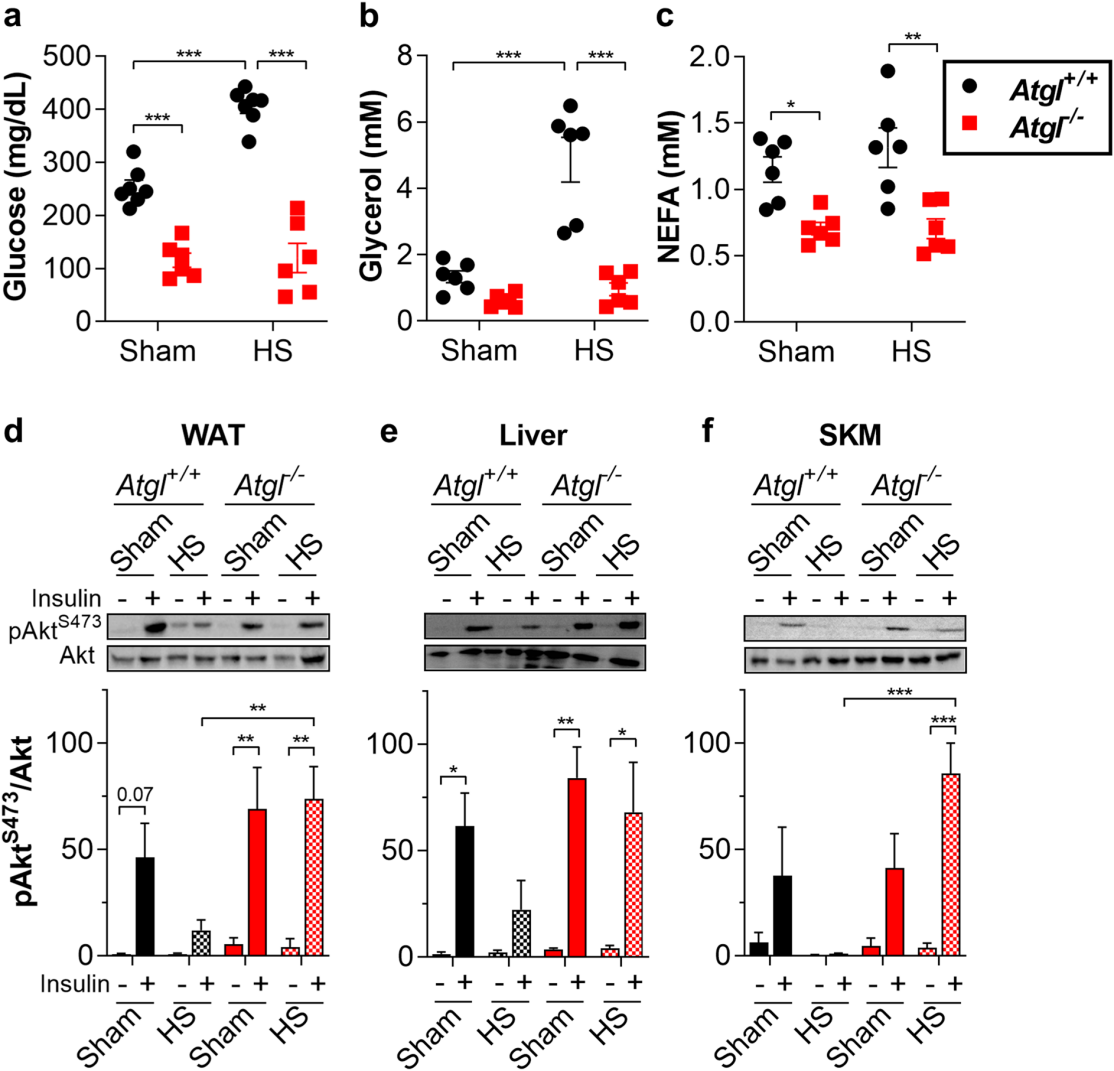
Global genetic ablation of ATGL improves insulin sensitivity during HS. (**a**) Glucose, (**b**) glycerol, and (**c**) NEFA levels in serum from *Atgl*^+/+^ and *Atgl*^−/−^ mice fasted 4 h then subjected to sham or HS. After 30 min of sham or HS, *Atgl*^+/+^ or *Atgl*^−/−^ mice were injected with saline or 0.5 U insulin in the inferior vena cava, and tissues were harvested after 4 min. Representative immunoblots and quantitation for (**d**) white adipose tissue (WAT, epididymal), (**e**) liver, and (**f**) skeletal muscle (SKM, gastrocnemius). *N* = 3–7. Data are shown as mean ± SEM. *P < 0.05, **P < 0.01, and ***P < 0.001, as indicated. For (**a**–**c**), statistical analyses were performed using two-way ANOVA. For (**d**–**f**), image quantitations were normalized by setting largest value in each immunoblot to 100. Statistical analyses on relative values were performed using three-way ANOVA. All comparisons differing by one variable were made and adjusted for with ANOVA, and all significant comparisons are denoted as such.

**Figure 2:**
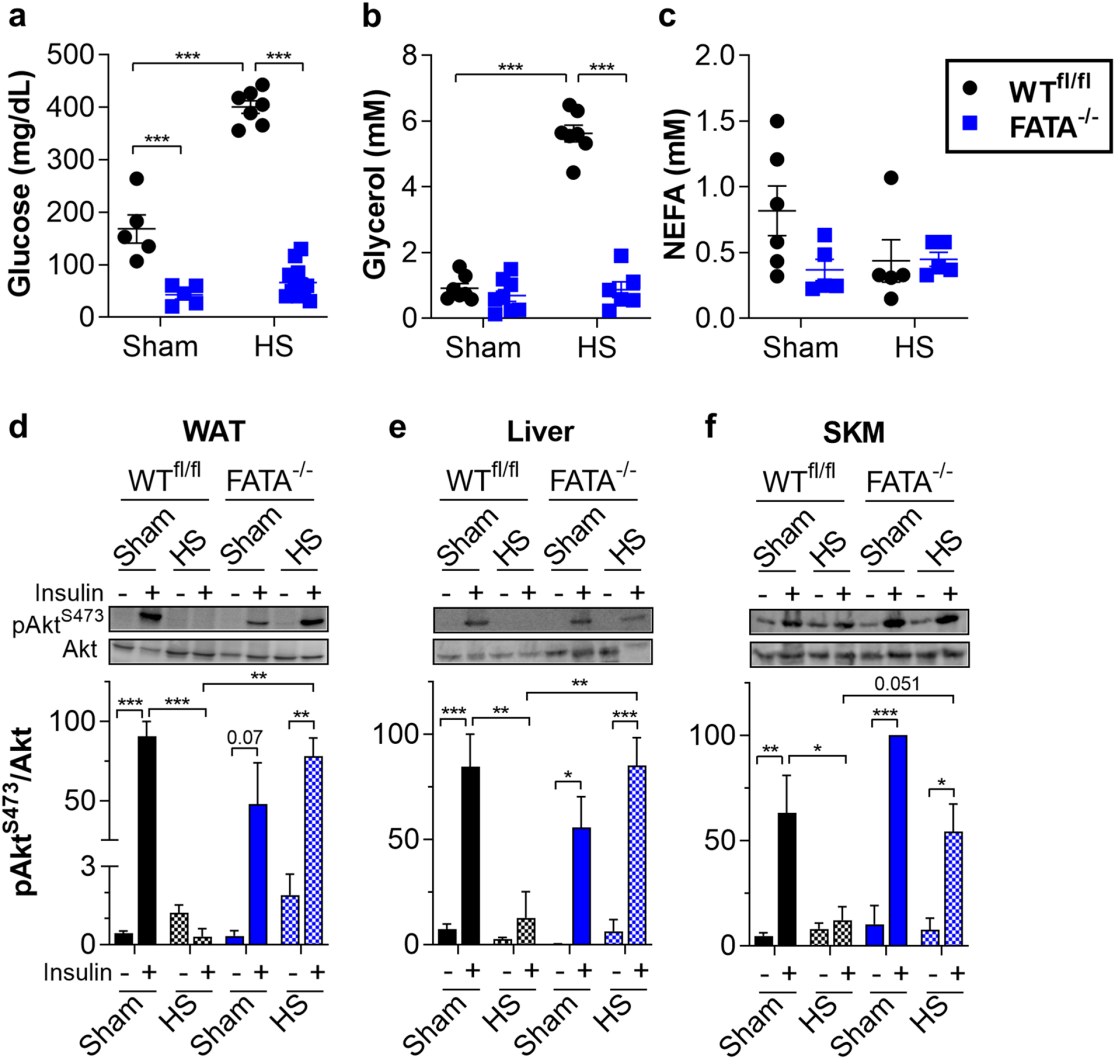
Genetic ablation of adipocyte ATGL improves insulin sensitivity during HS (**a**) glucose, (**b**) glycerol, and (**c**) NEFA levels in serum from WT^fl/fl^ and FATA^−/−^ mice fasted 4 h then subjected to sham or HS. After 30 min of sham or HS, WT^fl/fl^ or FATA^−/−^ mice were injected with saline or 0.5 U insulin in the inferior vena cava, and tissues were harvested after 4 min. Representative immunoblots and quantitation for (**d**) white adipose tissue (WAT, epididymal), (**e**) liver, and (**f**) skeletal muscle (SKM, gastrocnemius). *N* = 3–12. Data are shown as mean ± SEM. **P < 0.01 and ***P < 0.001, as indicated. For (**a**–**c**), statistical analyses were performed using two-way ANOVA. For (**d**–**f**), image quantitations were normalized by setting largest value in each immunoblot to 100. Statistical analyses on relative values were performed using three-way ANOVA. All comparisons differing by one variable were made and adjusted for with ANOVA, and all significant comparisons are denoted as such.

Rodent models of acute surgical injury and hemorrhage experience severe adrenergic stress and develop stress-induced metabolic derangements, including hyperglycemia and insulin resistance^1,21–23^. To investigate the requirement of lipolysis in mediating metabolic changes during acute stress, we subjected mice to soft tissue surgical injury alone (sham) or with HS. In wild-type (WT, *Atgl*^+/+^) mice, HS induced profound hyperglycemia (Fig. 1a), adipocyte TAG lipolysis as indicated by increased circulating glycerol (Fig. 1b), and hyperlactatemia (Supplementary Fig. S1a). These effects were abolished with inhibition of adipocyte TAG lipolysis in mice deficient for adipose triglyceride lipase (ATGL, *Atgl*^−/−^) (Fig. 1a, b, Supplementary Fig. S1a). Unlike glycerol, circulating NEFA levels were not elevated following HS (Fig. 1c). Consistent with a lack of elevation in NEFA, there was no apparent increase in ketone production (Supplementary Fig. S1b). HS impaired Akt^S473^ phosphorylation in peripheral tissues in response to insulin treatment (Fig. 1d–f), as previously reported^14,21,24^. Notably, *Atgl*^−/−^ mice were largely protected from HS-induced insulin resistance not only in WAT, but also in liver and skeletal muscle. To identify adipocytes as the necessary site of ATGL action, we genetically deleted *Atgl* specifically from adipocytes (FATA^−/−^). Successful adipose tissue-specific loss of ATGL was demonstrated by immunoblot (Supplementary Fig. S2a). The functional consequences of this deletion were confirmed by subjecting mice to i.p. injection of epinephrine and measuring the release of lipolytic products. Both serum glycerol and NEFA were observed to increase in WT mice, but this increase was prevented by genetic deletion of adipocyte ATGL in FATA^−/−^ mice (Supplementary Fig. S2b,c,g). To examine the role of adipocyte ATGL action in the context of acute stress, we subjected FATA^−/−^ mice to HS. Similar to global deletion, loss of ATGL in adipocytes alone lowered blood glucose levels and increased insulin sensitivity after HS (Fig. 2a,d–f).

Similar to the *Atgl*^*−/−*^ mice, the stimulation of adipose lipolysis in FATA^−/−^ mice was blocked as indicated by no induction of glycerol or NEFA with HS (Fig. 2b,c). Again, WT littermates showed induction of glycerol, but no corresponding increase in NEFA. This disconnect between circulating levels of the lipolytic products glycerol and NEFA has been previously ascribed to increased vasoconstriction in white adipose tissue (WAT) during HS that limits the availability of carrier proteins, such as albumin, for transport of NEFA into systemic circulation^25–29^. In the absence of transport out of the adipose depot, re-esterification of fatty acids is enhanced, an event that is exacerbated by elevated lactate and extracellular acidification. However, it is possible that our static NEFA measurement is missing flux from adipose to other peripheral tissues. To address the possibility of uptake by peripheral tissues, we measured TAG levels in liver and skeletal muscle. Liver TAGs were decreased in FATA^−/−^ mice compared to WT, corroborating previously published data (Supplementary Fig. S2d)^30^. However, there was no significant difference observed between sham and HS liver TAGs for WT mice. This combined with no significant changes in ketones suggests that substantial flux from adipose to liver is not responsible for the lack of change in static circulating levels (Supplementary Fig. S2h). In addition, no significant differences in TAG levels were observed in skeletal muscle (Supplementary Fig. S2e). To assess whether fatty acids are indeed trapped in adipose tissue by the lack transport into circulation, we measured NEFA levels within the epididymal adipose tissue (Supplementary Fig. S2f). Although there was a slight trend towards increased adipose tissue NEFA in WT mice under HS conditions, no significant changes were observed.

**Figure 3:**
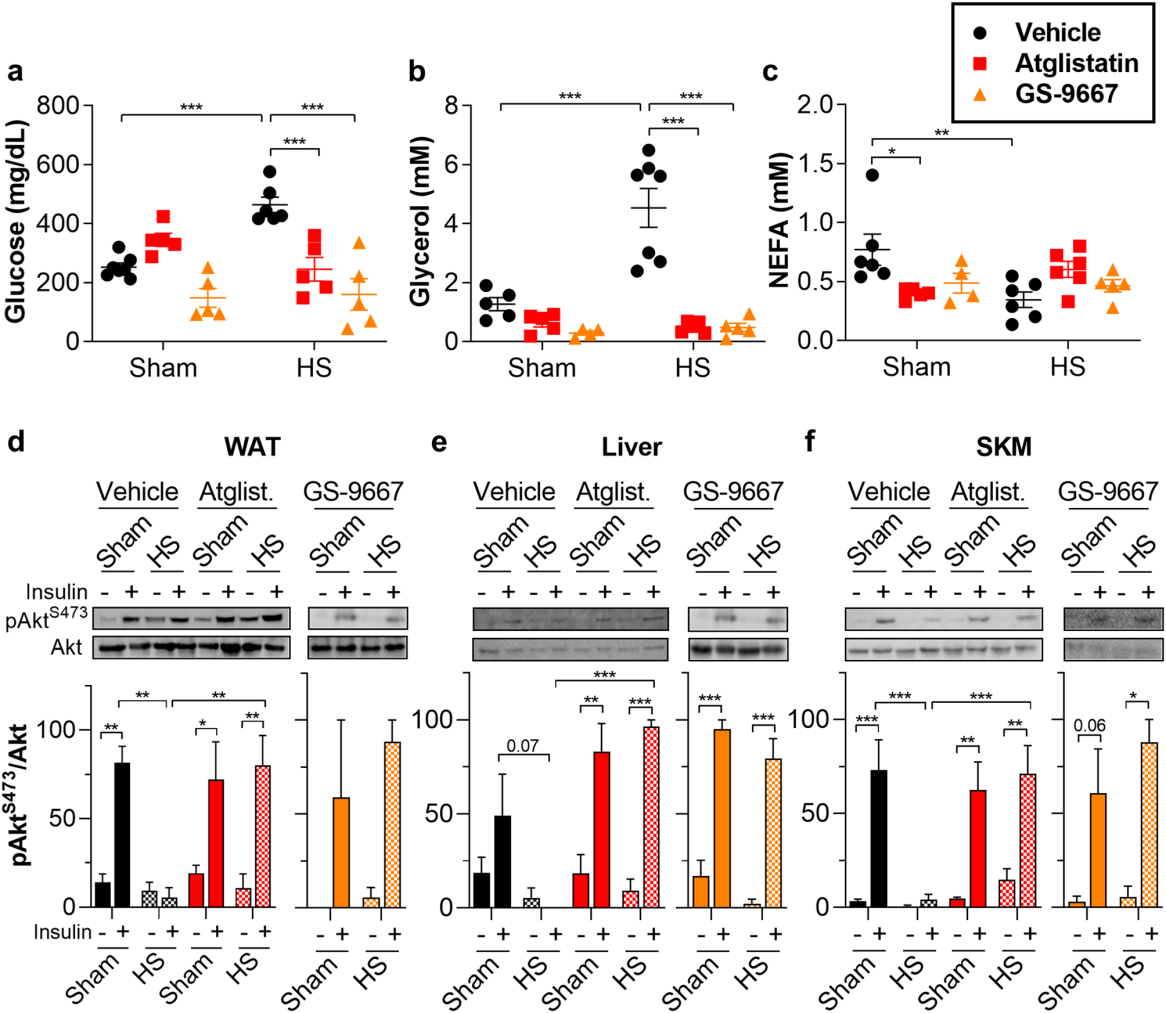
Pharmacological inhibition of lipolysis improves insulin sensitivity during HS. (**a**) Glucose, (**b**) glycerol, and (**c**) NEFA levels in serum from WT mice fasted 4 h; pretreated either with vehicle, 1 mg/kg Atglistatin, or 5 mg/kg GS-9667; and subjected to sham or HS. After 30 min of sham or HS, the mice were injected with saline or 0.5 U insulin in the inferior vena cava, and tissues were harvested after 4 min. Representative immunoblots and quantitation for (**d**) white adipose tissue (WAT, epididymal), (**e**) liver, and (**f**) skeletal muscle (SKM, gastrocnemius). *N* = 2–7. Data are shown as mean ± SEM. *P < 0.05, **P < 0.01, and ***P < 0.001, as indicated. For (**a**–**c**), statistical analyses were performed using two-way ANOVA. For (**d**–**f**), image quantitations were normalized by setting largest value in each immunoblot to 100. Statistical analyses on relative values for vehicle and atglistatin were performed using three-way ANOVA. Statistical analyses of GS-9667 relative values were performed using two-way ANOVA All comparisons differing by one variable were made and adjusted for with ANOVA, and all significant comparisons are denoted as such.

We next used pharmacological treatment to acutely inhibit lipolysis. Atglistatin is a specific ATGL inhibitor^31^. Similar to genetic loss of ATGL, Atglistatin attenuated hyperglycemia (Fig. 3a) and lipolysis (Fig. 3b–c, Supplementary Fig. S3a) and also improved insulin sensitivity (Fig. 3d–f) after HS. While Atglistatin is a potent inhibitor of rodent ATGL, it is ineffective against the human isoform^32^. Therefore, we chose to confirm our results with GS-9667, an adenosine A1 receptor (A1-AR) partial agonist that blocks adipocyte lipolysis by inhibiting cAMP production. GS-9667 has been shown to be effective in reducing circulating NEFA in human patients^33,34^. We found that pretreatment with GS-9667 was also effective at preventing HS-induced insulin resistance and hyperglycemia (Fig. 3a–f, Supplementary Fig. S3a). These studies suggest that stress-induced insulin resistance and hyperglycemia are dependent on adipocyte TAG lipolysis but occur without apparent increases in circulating NEFA.

### Blocking adipocyte glycerol release reduces acute adrenergic stress-induced hyperglycemia

Stress hormone action on liver rapidly mobilizes liver glycogen stores to raise circulating glucose^22^. To test whether liver glycogenolysis is responsible for stress-induced hyperglycemia, we examined glycogen levels in our models of abrogated lipolysis. Consistent with previous findings, HS induced a significant reduction of hepatic glycogen levels (Supplementary Fig. S4a-c) in WT mice when compared with sham controls. However, mice with global or adipocyte-specific deletion of *Atgl* exhibited lower liver glycogen levels than WT controls under sham conditions (Supplementary Fig. S4a-b). Previous reports also found rapid depletion of liver glycogen, even during short-term fasts such as those undergone by mice in our experiments, in global and adipocyte-specific *Atgl*-knockout mice^30,35^. Perhaps to compensate for decreased glycogen availability^30,36^, the FATA^−/−^ mice elevated hepatic gluconeogenic gene expression (Supplementary Table S1). However, unlike ATGL genetic deletion, acute inhibition of lipolysis by Atglistatin or GS-9667 preserved liver glycogen levels under sham conditions (Supplementary Fig. S4c) and, notably, still attenuated hyperglycemia. Taken together, these data highlight that glycogenolysis does not wholly explain stress-induced hyperglycemia and that adipocyte TAG lipolysis is required for increased glucose output during acute stress.

Additionally, stress hormone action, epinephrine in particular, is known to induce adipocyte lipolysis to increase the availability of glycerol for liver gluconeogenesis. We next investigated the specific contribution of the lipolytic product glycerol to stress hyperglycemia. Glycerol enters the circulation after efflux through Aquaporin 7 (AQP7), the major glycerol release channel in adipocytes. Mice deficient for AQP7 (*Aqp7*^−/−^) cannot release glycerol into circulation in response to adrenergic stimulation^17,37^ and were protected from hyperglycemia during HS (Supplementary Fig. S5a–e). Notably, *Aqp7*^*−/−*^ mice had similar liver glycogen levels as *Aqp7*^+/+^ under sham conditions (Supplementary Fig. S5f). *Aqp7*^−/−^ mice are still capable of breaking down TAG in response to stimulated lipolysis. However, developmental adaptations in these mice increases glycerol kinase activity in adipocytes, leading to enhanced fatty acid re-esterification and a reduced net lipolysis^17,37^. Likely due to the reduction in lipolysis, *Aqp7*^−/−^ mice maintained WAT and skeletal muscle insulin sensitivity during HS, and hepatic insulin signaling was partially rescued, though not statistically significant (Supplementary Fig. S5g–i).

Administration of glycerol to vehicle- or Atglistatin-treated mice to achieve levels normally observed during HS substantially increased glucose levels (Supplementary Fig. S6a-c). This suggests that glycerol alone is sufficient to induce the same degree of hyperglycemia in sham or Atglistatin-treated HS mice. However, this did not normalize peripheral insulin signaling (Supplementary Fig. S6g), nor did the glycerol-mediated increase in glucose levels prevent the elevated catecholamine or insulin levels induced by Atglistatin during HS (Supplementary Fig. S6d–f). These studies show that glycerol is an important contributor to acute stress-induced hyperglycemia but does not explain the development of peripheral insulin resistance.

### Lipolysis inhibition does not blunt the sympathetic response to stress

We next investigated whether inhibition of lipolysis altered the counterregulatory response to stress as a possible explanation for improved insulin action. Circulating levels of norepinephrine were similar in sham and HS mice (Supplementary Fig. S7a–d). Corticosterone was slightly elevated by HS regardless of lipolysis inhibition (Supplementary Fig. S7e–h). In response to HS, epinephrine levels showed an acute induction and increased further with genetic or pharmacologic inhibition of lipolysis (Supplementary Fig. S7i–l), as previously observed^8,38^. HS-induced insulin levels were potentiated with lipolysis inhibition (Supplementary Fig. S7m–p)^21^, except during GS-9667 treatment (Supplementary Fig. S7o). The latter effect may be due to GS-9667 action on A1-AR of pancreatic β-cells, thereby interfering with insulin release^8,39^. These data show that inhibition of lipolysis improves insulin sensitivity without blunting the counterregulatory stress response and despite higher epinephrine levels.

### Lipolysis inhibition improves peripheral glucose uptake and EGP during catecholamine stimulation

**Figure 4:**
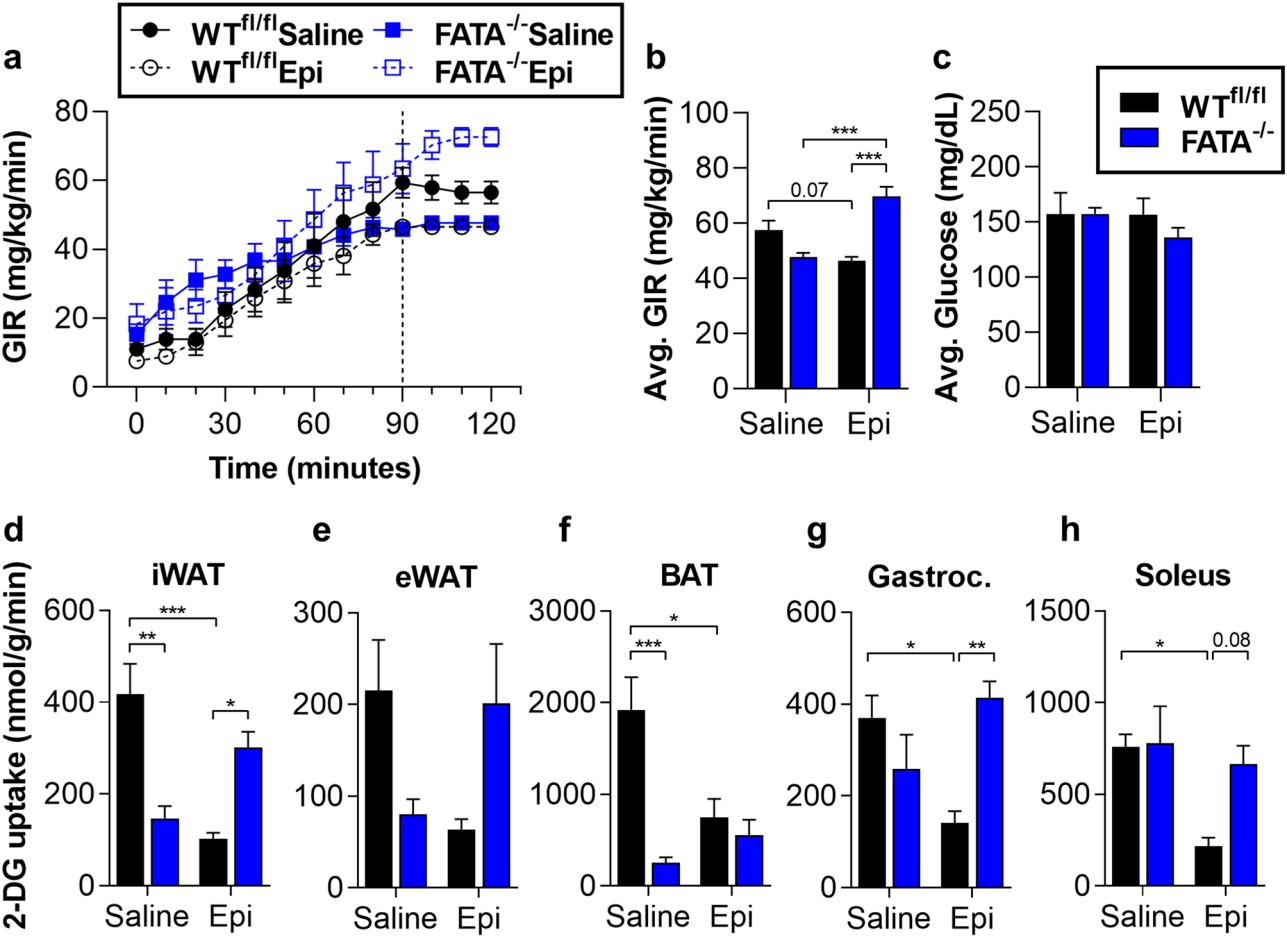
Genetic deletion of adipocyte ATGL abolishes epinephrine-induced EGP and impaired glucose uptake during hyperinsulinemic–euglycemic clamps. WT^fl/fl^ or FATA^−/−^ mice fasted 2–4 h were infused with either saline or epinephrine (Epi, 1 µg/kg/min) and subjected to a 30 min basal period (− 30 to 0 min) followed by a hyperinsulinemic–euglycemic clamp (0–120 min). The mice then received a bolus of 2-DG, and the clamp was continued for an additional 30 min (120–150 min). (**a**) Glucose infusion rate during the course of the clamp. Dashed line indicates beginning of steady state (90–120 min). (**b**) Average glucose infusion rates (GIR) and (**c**) average blood glucose during steady state. 2-DG uptake in (**d**) inguinal white adipose tissue (iWAT), (**e**) epididymal white adipose tissue (eWAT), (**f**) brown adipose tissue (BAT), (**g**) gastrocnemius (Gastroc.) muscle, and (**h**) soleus muscle of WT^fl/fl^ or FATA^−/−^ mice following hyperinsulinemic–euglycemic clamp. *N* = 4–7. Data are shown as mean ± SEM. *P < 0.05, **P < 0.01, and ***P < 0.001, as indicated. Statistical analyses were performed using two-way ANOVA. All comparisons differing by one variable were made and adjusted for with ANOVA, and all significant comparisons are denoted as such.

While the degree of Akt phosphorylation under these experimental conditions suggests insulin resistance in agreement with previous results^14,21,24^, we were interested in making a more quantitative assessment of insulin sensitivity in peripheral tissues. We have previously shown that ATGL activity is required for catecholamine-induced impairment of glucose uptake in isolated adipocytes^16^. To investigate the role of stress-induced adipocyte lipolysis on glucose uptake in vivo under controlled conditions, we infused epinephrine in anesthetized WT^fl/fl^ or FATA^−/−^ mice during hyperinsulinemic–euglycemic clamps^19^. As expected, WT^fl/fl^ mice exhibited an epinephrine-dependent decline in the glucose infusion rate (GIR) (Fig. 4a–c, Supplementary Fig. S8a)^7,40^. Epinephrine decreased insulin-stimulated glucose uptake by skeletal muscle and adipose tissues (Fig. 4d–h), without increasing circulating NEFA over saline controls (Supplementary Fig. S8b,c). Genetic inhibition of adipocyte lipolysis in FATA^−/−^ mice prevented epinephrine-induced impairments in glucose uptake (Fig. 4d–h), with hormone levels consistent between genotypes (Supplementary Fig. S8d–g).

**Figure 5:**
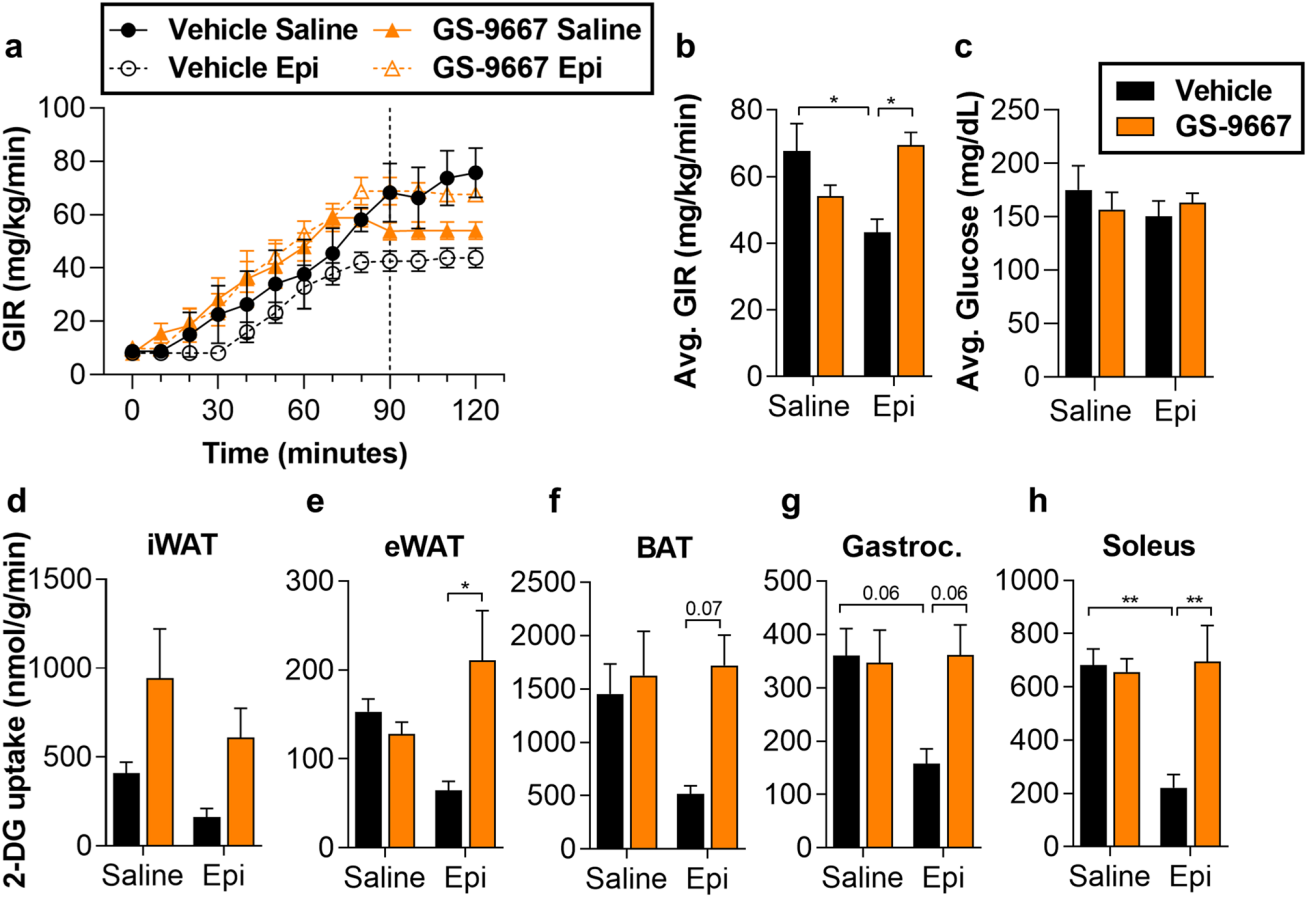
Pharmacological inhibition of adipocyte lipolysis reverses epinephrine (Epi)-induced EGP and impaired glucose uptake during hyperinsulinemic–euglycemic clamps. WT mice fasted 4 h and pretreated 30 min prior with vehicle or 5 mg/kg GS-9667. Mice were infused with either saline or epinephrine (Epi, 1 µg/kg/min) and subjected to a 30 min basal period (− 30 to 0 min) followed by a hyperinsulinemic–euglycemic clamp (0–120 min). The mice then received a bolus of 2-DG, and the clamp was continued for an additional 30 min (120–150 min). (**a**) Glucose infusion rate during the course of the clamp. Dashed line indicates beginning of steady state (90–120 min). (**b**) Average glucose infusion rates (GIR) and (**c**) average blood glucose during steady state. 2-DG uptake in (**d**) inguinal white adipose tissue (iWAT), (**e**) epididymal white adipose tissue (eWAT), (**f**) brown adipose tissue (BAT), (**g**) gastrocnemius (Gastroc.) muscle, and (**h**) soleus muscle of vehicle- or GS-9667-treated mice following hyperinsulinemic–euglycemic clamp. *N* = 4–5. Data are shown as mean ± SEM. *P < 0.05 and **P < 0.01, as indicated. Statistical analyses were performed using two-way ANOVA. All comparisons differing by one variable were made and adjusted for with ANOVA, and all significant comparisons are denoted as such.

Similar to what was observed in the FATA^−/−^ mice, pharmacological inhibition of lipolysis by GS-9667 improved the decreased GIR observed after epinephrine infusion and restored insulin sensitivity in skeletal muscle and adipose tissue (Fig. 5, Supplementary Fig. S9a–c). Epinephrine infusion did not alter the levels of other stress hormones (Supplementary Fig. S9d–g). Thus, in accordance with previous findings, adipocyte lipolysis regulates glucose production during the stress response^7,8,22^. Further, we see that the significant impairments in tissue-specific glucose uptake induced by epinephrine are reversed by inhibiting adipocyte lipolysis (Fig. 5d–h).

### Resistin shows a positive correlation with insulin resistance, in a lipolysis dependent manner

**Figure 6:**
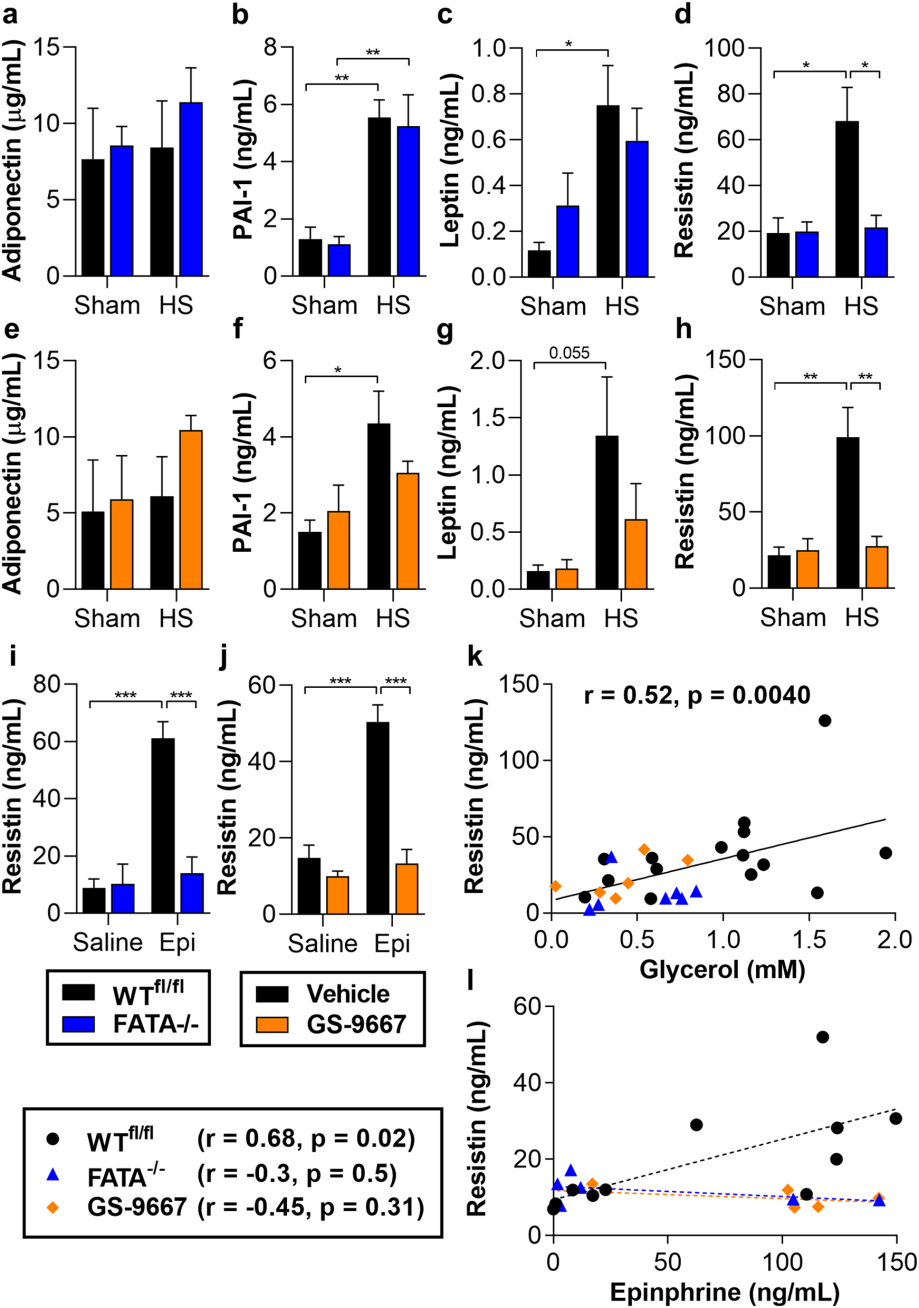
Resistin levels correlate with lipolysis and insulin resistance during HS. (**a**) Adiponectin, (**b**) PAI-1, (**c**) leptin, and (**d**) resistin in serum from WT^fl/fl^ or FATA^−/−^ mice subjected to sham or HS for 30 min. (**e**–**h**) As in (**a**–**d**), but in mice pretreated with vehicle or GS-9667 before HS. (**i**) Resistin levels in WT^fl/fl^ and FATA^−/−^ mice at conclusion of hyperinsulinemic–euglycemic clamp with saline or epinephrine (Epi) infusion. (**j**) As in (**i**), but from WT mice pretreated with vehicle or GS-9667 before clamp. (**k**) Correlation (Pearson) between circulating resistin and glycerol from mice subjected to sham or HS with and without genetic or pharmacological inhibition of lipolysis. (**l**) Correlation (Pearson) between circulating resistin and epinephrine from mice subjected to hyperinsulinemic–euglycemic clamp with saline or epinephrine infusion, in WT (black dotted line), FATA^−/−^ (blue dotted line), and GS-9667-treated (orange dotted line) mice. *N* = 3–6. Data are shown as mean ± SEM. *P < 0.05, **P < 0.01, and ***P < 0.001, as indicated. Statistical analyses were performed using two-way ANOVA. All comparisons differing by one variable were made and adjusted for with ANOVA, and all significant comparisons are denoted as such.

In addition to storing and mobilizing TAG, adipocytes also release cytokines and adipokines. Proinflammatory cytokines have been implicated in mediating insulin resistance during chronic WAT inflammation in obesity and NASH^41^. However, circulating proinflammatory cytokines did not show consistent differences with inhibition of lipolysis during acute stress (Supplementary Tables S2, S3). Adipokines such as adiponectin and leptin are linked to improved insulin sensitivity^42,43^. Adiponectin levels were unaffected by HS or inhibition of lipolysis, while circulating levels of plasminogen activator inhibitor-1 (PAI-1) and leptin were either elevated by HS or trended towards increased but in a lipolysis-independent manner (Fig. 6a–c,e–g). Resistin is a pro-inflammatory adipokine that positively correlates with hyperglycemia and insulin resistance in obesity, and both peripheral and hypothalamic administrations of resistin impair insulin signaling^44–46^. Interestingly, resistin showed a robust induction with HS, which was attenuated with inhibition of lipolysis (Fig. 6d,h). Similarly, resistin levels failed to increase with HS in *Aqp7*^−/−^ mice (Supplementary Table S4). In addition, epinephrine infusion increased circulating resistin levels, and simultaneous lipolysis inhibition abrogated this response (Fig. 6i,j). Circulating resistin levels were positively correlated with net lipolysis as shown by glycerol levels in mice subjected to Sham or HS (Fig. 6k). And circulating resistin showed a lipolysis-dependent, positive correlation with epinephrine levels during clamps (Fig. 6l). Numerous clinical studies observed correlations between circulating resistin levels and the development of stress-induced metabolic dysfunction and morbidity^47^. While not providing definitive mechanistic evidence, our data does corroborate a strong relationship between resistin and stress-induced insulin resistance and support a lipolysis-mediated resistin release during acute stress.

## Discussion

This study shows that adrenergic action and subsequent adipocyte lipolysis is necessary to drive stress-induced impairments in peripheral glucose disposal and whole body insulin resistance. Our observations put adipose tissue forward as a central player in the metabolic response to acute stress and challenge the current paradigms that stress-induced global insulin resistance and hyperglycemia arise from direct effects of counterregulatory hormones on multiple tissues.

Glycerol is an important gluconeogenic precursor. Under basal conditions glycerol likely contributes little to gluconeogenesis, however under stress conditions, as during HS, the need for glycerol can rise dramatically^48^. Here, we show that preventing the release of glycerol into circulation attenuates HS-induced hyperglycemia. Furthermore, infusion of glycerol during suppression of lipolysis significantly elevated blood glucose levels during acute stress. These findings indicate that glycerol derived from adipocyte lipolysis is an important contributor to stress-induced hyperglycemia.

While trauma and injury induce a strong counterregulatory response, HS significantly upregulates catecholamines, particularly epinephrine^22,49^. Epinephrine reduces whole body glucose disposal by increasing endogenous glucose release and inhibiting glucose uptake primarily in skeletal muscle. Epinephrine stimulates EGP by promoting hepatic glycogenolysis and gluconeogenesis through direct and indirect effects on liver, respectively^7,8^. McGuinness and colleagues showed that epinephrine played a pivotal role in regulating EGP during stress hormone infusion^22,23,50^. Other studies have demonstrated the well-known effect of insulin to suppress hepatic gluconeogenesis was an indirect effect dependent on inhibition of adipocyte lipolysis by insulin^8^. Indeed, preventing the fall of circulating NEFA during fasting was sufficient to maintain elevated gluconeogenesis, and inhibiting adipocyte lipolysis led to a reduction in EGP independent of hepatic insulin signaling^9^. Our data demonstrate that attenuating adipocyte lipolysis is sufficient to block HS or acute epinephrine-induced impairments in the rates of glucose disappearance and are in agreement with these earlier studies.

While the mechanisms underlying increased EGP in response to epinephrine are well understood, it is less clear how epinephrine impairs glucose disposal. While exogenously infused NEFA inhibits peripheral glucose uptake, this occurs after several hours delay^51^, far longer than the rapid onset of insulin resistance seen in the HS model. Studies sampling blood in veins outflowing both visceral and subcutaneous adipose depots found that glycerol, but not NEFA, was increased by HS^27,29^. Consistent with previous reports, we find that HS induces lipolysis but does not increase the level of circulating NEFA. In addition to vasoconstriction, hemorrhage also leads to elevated lactate levels and tissue acidification, thereby promoting increased fatty acid re-esterification within the adipose depot^25^. Circulating levels of the ketone body β-hydroxybuytyrate (β-OHB) mirrored NEFA levels, suggesting that NEFA flux from adipocytes to liver was decreased. Thus, while adipocyte lipolysis is required for the induction of insulin resistance and impaired glucose uptake during acute stress, our studies suggest this could be uncoupled from an elevation in circulating NEFA. However, an important caveat to examining static levels of NEFA in circulation is that this could miss an increase in NEFA flux from adipocytes into tissues. Until studies examining lipid flux under these conditions have been performed, we cannot rule out the possibility that release of NEFA from adipose into circulation has been missed due to compensating changes in uptake or oxidation.

Adipose tissue is not only an important regulator of energy homeostasis, but it also modulates insulin-stimulated peripheral body glucose disposal by multiple mechanisms. In addition to NEFA-induced lipotoxicity, adipose-derived factors such as adipokines, cytokines, and lipid intermediates impair insulin-stimulated glucose disposal^52^. For example, the adipokine resistin is elevated in obesity and type 2 diabetes^46,53^. In mice, resistin is released by adipocytes, whereas in humans it is secreted from activated macrophages. However, mice that secrete ‘humanized’ resistin from macrophages also develop insulin resistance^54^. Recent studies also found increased circulating resistin levels in critically ill patients and observed a strong positive correlation between resistin levels and mortality^47,55–57^. In our mouse model of acute stress, we consistently observed increases in circulating levels of resistin with HS compared to sham. This reflects the results of human studies examining patients experiencing spontaneous acute basal ganglia hemorrhage, traumatic subarachnoid hemorrhage, and hemorrhagic fever, where resistin levels were found to be substantially elevate and positively correlated with hemorrhage, insulin resistin, and mortality^58–60^. Furthermore, our data demonstrate for the first time that resistin release requires lipolysis, and the lipolysis-dependent induction in our study strongly correlated with insulin resistance and impaired glucose disposal. Thus, the potential role of resistin in stress-induced peripheral insulin resistance during injury shows promise and should be the subject of further study.

One limitation of our study is the short time frame that was investigated. While catecholamines are very important during the initial response to injury and stress, other hormones play significant roles in the days to weeks following injury. This includes cortisol, inflammatory cytokines, and glucagon. They are induced at different times during the stress response and have tissue-specific effects. It is important to note that all of these hormones and cytokines may act similarly; they all oppose insulin action and induce adipocyte lipolysis. By focusing on one important component of the stress response in the acute setting, we have been able to define adipocyte lipolysis as a key link to impaired metabolism in response to catecholamine action. This sets the stage for further investigations into more chronic conditions. Another limitation is that these experiments were performed under anesthesia. While appropriate for comparisons with patients undergoing invasive surgeries, anesthesia is well known to impact insulin sensitivity^61,62^.

In summary, using both genetic and pharmacological models we show that adipocyte TAG lipolysis is required for the development of hemorrhage- and epinephrine-induced hyperglycemia and insulin resistance. Our data suggest that the improved glycemia and insulin-sensitivity observed with inhibition of lipolysis may involve decreased resistin release. These findings have important implications for understanding the early metabolic changes in the development of stress hyperglycemia during physical injury and HS commonly encountered in surgical and critically ill patients. Our studies highlight a key role for the adipocyte in regulating stress-induced metabolic dysfunction in other tissues through crosstalk and may open up new avenues to improve glucose control and insulin sensitivity after trauma and surgery.

## Supplementary information

Supplementary Information

## Data Availability

All data generated or analyzed during this study are included in the published article (and its online supplementary files). Raw data is available on request. The resources (animals) that support the findings of this study are available from the Jackson labs (Bar Harbor, ME) and the Riken Resource Center (Japan).

## References

[1] Chaudry IH, Sayeed MM, Baue AE (1974). Insulin resistance in experimental shock. Arch. Surg..

[2] Li L, Messina JL (2009). Acute insulin resistance following injury. Trends Endocrinol. Metab..

[3] Duggan EW, Carlson K, Umpierrez GE (2017). Perioperative hyperglycemia management: An update. Anesthesiology.

[4] Ljungqvist O, Jonathan E (2012). Rhoads lecture 2011: Insulin resistance and enhanced recovery after surgery. J. Parent. Enteral Nutr..

[5] Lipshutz AK, Gropper MA (2009). Perioperative glycemic control: An evidence-based review. Anesthesiology.

[6] Vanhorebeek I, Gunst J, Van den Berghe G (2018). Critical care management of stress-induced hyperglycemia. Curr. Diab.Rep..

[7] Chu CA (1997). Comparison of the direct and indirect effects of epinephrine on hepatic glucose production. J. Clin. Investig..

[8] Mittelman SD, Bergman RN (2000). Inhibition of lipolysis causes suppression of endogenous glucose production independent of changes in insulin. American j. Physiol. Endocrinol. Metab..

[9] Perry RJ (2015). Hepatic acetyl CoA links adipose tissue inflammation to hepatic insulin resistance and type 2 diabetes. Cell.

[10] Titchenell PM (2016). Direct hepatocyte insulin signaling is required for lipogenesis but is dispensable for the suppression of glucose production. Cell Metab..

[11] Pontzer H (2016). Metabolic acceleration and the evolution of human brain size and life history. Nature.

[12] Abel ED (2001). Adipose-selective targeting of the GLUT4 gene impairs insulin action in muscle and liver. Nature.

[13] Kumar A (2010). Fat cell-specific ablation of rictor in mice impairs insulin-regulated fat cell and whole-body glucose and lipid metabolism. Diabetes.

[14] Williams VL, Martin RE, Franklin JL, Hardy RW, Messina JL (2012). Injury-induced insulin resistance in adipose tissue. Biochem. Biophys. Res. Commun..

[15] Li L, Thompson LH, Zhao L, Messina JL (2009). Tissue-specific difference in the molecular mechanisms for the development of acute insulin resistance after injury. Endocrinology.

[16] Mullins GR (2014). Catecholamine-induced lipolysis causes mTOR complex dissociation and inhibits glucose uptake in adipocytes. Proc. Natl. Acad. Sci. USA.

[17] Maeda N (2004). Adaptation to fasting by glycerol transport through aquaporin 7 in adipose tissue. Proc. Natl. Acad. Sci. USA.

[18] Zhai L, Ballinger SW, Messina JL (2011). Role of reactive oxygen species in injury-induced insulin resistance. Mol. Endocrinol..

[19] Ayala JE (2011). Hyperinsulinemic–euglycemic clamps in conscious, unrestrained mice. J. Vis. Exp..

[20] Hoehn KL (2009). Insulin resistance is a cellular antioxidant defense mechanism. Proc. Natl. Acad. Sci. USA.

[21] Ma Y, Wang P, Kuebler JF, Chaudry IH, Messina JL (2003). Hemorrhage induces the rapid development of hepatic insulin resistance. Am. J. Physiol. Gastrointest. Liver Physiol..

[22] Stevenson RW (1991). Dose-related effects of epinephrine on glucose production in conscious dogs. Am. J. Physiol..

[23] McGuinness OP (1993). Impact of chronic stress hormone infusion on hepatic carbohydrate metabolism in the conscious dog. Am. J. Physiol..

[24] Thompson LH, Kim HT, Ma Y, Kokorina NA, Messina JL (2008). Acute, muscle-type specific insulin resistance following injury. Mol. Med..

[25] Rosell S, Belfrage E (1979). Blood circulation in adipose tissue. Physiol. Rev..

[26] Belfrage E, Hjemdahl P, Fredholm BB (1979). Metabolic effects of blood flow restriction in adipose tissue. Acta Physiol. Scand..

[27] Kovach AG (1970). Blood flow, oxygen consumption, and free fatty acid release in subcutaneous adipose tissue during hemorrhagic shock in control and phenoxybenzamine-treated dogs. Circ. Res..

[28] Rofe AM, Williamson DH (1983). Mechanism for the 'anti-lipolytic' action of vasopressin in the starved rat. Biochem. J..

[29] Kovach AG (1974). Blood flow and release of free fatty acids in the omentum, mesentery and subcutaneous adipose tissue of the dog in haemorrhagic shock. Acta Physiol. Acad. Sci. Hung..

[30] Schoiswohl G (2015). Impact of reduced ATGL-mediated adipocyte lipolysis on obesity-associated insulin resistance and inflammation in male mice. Endocrinology.

[31] Mayer N (2013). Development of small-molecule inhibitors targeting adipose triglyceride lipase. Nat. Chem. Biol..

[32] Schweiger M (2017). Pharmacological inhibition of adipose triglyceride lipase corrects high-fat diet-induced insulin resistance and hepatosteatosis in mice. Nat. Commun..

[33] Dhalla AK, Wong MY, Voshol PJ, Belardinelli L, Reaven GM (2007). A1 adenosine receptor partial agonist lowers plasma FFA and improves insulin resistance induced by high-fat diet in rodents. Am. J. Physiol. Endocrinol. Metab..

[34] Staehr PM (2013). Reduction of free fatty acids, safety, and pharmacokinetics of oral GS-9667, an A(1) adenosine receptor partial agonist. J. Clin. Pharmacol..

[35] Schoiswohl G (2010). Adipose triglyceride lipase plays a key role in the supply of the working muscle with fatty acids. J. Lipid Res..

[36] Kienesberger PC (2009). Adipose triglyceride lipase deficiency causes tissue-specific changes in insulin signaling. J. Biol. Chem..

[37] Hibuse T (2005). Aquaporin 7 deficiency is associated with development of obesity through activation of adipose glycerol kinase. Proc. Natl. Acad. Sci. USA.

[38] Chen X, Iqbal N, Boden G (1999). The effects of free fatty acids on gluconeogenesis and glycogenolysis in normal subjects. J. Clin. Investig..

[39] Topfer M, Burbiel CE, Muller CE, Knittel J, Verspohl EJ (2008). Modulation of insulin release by adenosine A1 receptor agonists and antagonists in INS-1 cells: The possible contribution of 86Rb+ efflux and 45Ca2+ uptake. Cell Biochem. Funct..

[40] Deibert DC, DeFronzo RA (1980). Epinephrine-induced insulin resistance in man. J. Clin. Investig..

[41] Glass CK, Olefsky JM (2012). Inflammation and lipid signaling in the etiology of insulin resistance. Cell Metab..

[42] Perry RJ (2018). Leptin mediates a glucose-fatty acid cycle to maintain glucose homeostasis in starvation. Cell.

[43] Berg AH, Combs TP, Du X, Brownlee M, Scherer PE (2001). The adipocyte-secreted protein Acrp30 enhances hepatic insulin action. Nat. Med..

[44] Muse ED, Lam TK, Scherer PE, Rossetti L (2007). Hypothalamic resistin induces hepatic insulin resistance. J. Clin. Investig..

[45] Muse ED (2004). Role of resistin in diet-induced hepatic insulin resistance. J. Clin. Investig..

[46] Rajala MW (2004). Regulation of resistin expression and circulating levels in obesity, diabetes, and fasting. Diabetes.

[47] Hajri T, Gharib M, Kaul S, Karpeh MS (2017). Association between adipokines and critical illness outcomes. J. Trauma Acute Care Surg..

[48] Jahoor F, Klein S, Wolfe R (1992). Mechanism of regulation of glucose production by lipolysis in humans. Am. J. Physiol..

[49] Watts DT (1956). Arterial blood epinephrine levels during hemorrhagic hypotension in dogs. Am. J. Physiol..

[50] McGuinness OP (1999). Impact of acute epinephrine removal on hepatic glucose metabolism during stress hormone infusion. Metab. Clin. Exp..

[51] Bonadonna RC, Zych K, Boni C, Ferrannini E, DeFronzo RA (1989). Time dependence of the interaction between lipid and glucose in humans. Am. J. Physiol..

[52] Petersen MC, Shulman GI (2018). Mechanisms of insulin action and insulin resistance. Physiol. Rev..

[53] Steppan CM (2001). The hormone resistin links obesity to diabetes. Nature.

[54] Qatanani M, Szwergold NR, Greaves DR, Ahima RS, Lazar MA (2009). Macrophage-derived human resistin exacerbates adipose tissue inflammation and insulin resistance in mice. J. Clin. Investig..

[55] Sunden-Cullberg J (2007). Pronounced elevation of resistin correlates with severity of disease in severe sepsis and septic shock. Crit. Care Med..

[56] Macdonald SP (2014). Sustained elevation of resistin, NGAL and IL-8 are associated with severe sepsis/septic shock in the emergency department. PLoS One.

[57] Koch A, Gressner OA, Sanson E, Tacke F, Trautwein C (2009). Serum resistin levels in critically ill patients are associated with inflammation, organ dysfunction and metabolism and may predict survival of non-septic patients. Crit. Care.

[58] Dong XQ, Hu YY, Yu WH, Zhang ZY (2010). High concentrations of resistin in the peripheral blood of patients with acute basal ganglia hemorrhage are associated with poor outcome. J. Crit. Care.

[59] Dong XQ (2010). Resistin is associated with mortality in patients with traumatic brain injury. Crit. Care.

[60] Erturk A (2014). Serum resistin levels may be new prognostic factor of crimean-congo hemorrhagic fever. Int J Clin Exp Med.

[61] Windelov JA, Pedersen J, Holst JJ (2016). Use of anesthesia dramatically alters the oral glucose tolerance and insulin secretion in C57Bl/6 mice. Physiol. Rep..

[62] Horber FF (1990). Isoflurane and whole body leucine, glucose, and fatty acid metabolism in dogs. Anesthesiology.

