# Supplementary material for: Adipocyte lipolysis drives acute stress-induced insulin resistance
Source: Sci Rep. 2020 Oct 23;10:18166. doi: 10.1038/s41598-020-75321-0 (PMC7584576; doi:10.1038/s41598-020-75321-0)
Supplement: Supplementary file 1 — Supplementary Information [file 41598_2020_75321_MOESM1_ESM.pdf]

SUPPLEMENTARY DATA

Adipocyte lipolysis drives acute stress-induced insulin resistance

Authors: Vidisha Raje[1], Katelyn W. Ahern[1], Brittany A. Martinez[1], Nancy L. Howell[2], Vici Oenarto[1,4], Mitchell E. Granade[1], Jae Woo Kim[1], Smanla Tundup[3], Katharina Bottermann[1], Axel Gödecke[4], Susanna R. Keller[2], Alexandra Kadl[1,3], Michelle L. Bland[1], Thurl E. Harris[1*]

Affiliations:

1. Department of Pharmacology, University of Virginia, Charlottesville, VA
2. Department of Medicine, Endocrinology and Metabolism, University of Virginia, Charlottesville, VA
3. Department of Medicine, Pulmonary and Critical Care Medicine, University of Virginia, Charlottesville, VA
4. Institute of Cardiovascular Physiology, Heinrich Heine University Düsseldorf, Germany

Supplementary Materials

1. **Supplementary Figures S1- S9**

Figure S1: Effect of genetic inhibition of global lipolysis on lactate and ketones.

Figure S2: Adipocyte-specific ATGL knockout and effect of genetic inhibition of adipocyte lipolysis on metabolite levels.

Figure S3: Pharmacologic inhibition of lipolysis does not impact ketone production.

Figure S4: Glycogen concentrations following HS.

Figure S5: Glycerol release during HS contributes to hyperglycemia.

Figure S6: Glycerol infusion causes hyperglycemia in the absence of lipolysis.

Figure S7: Counterregulatory hormone and insulin levels during HS.

Figure S8: Glucose infusion rate, hormone and lipolytic products in hyperinsulinemic-euglycemic clamps with epinephrine infusion in $FATA^{-/-}$ mice.

Figure S9: Glucose infusion rate, hormone and lipolytic products in hyperinsulinemic-euglycemic clamps with epinephrine infusion after GS-9667 treatment.

2. **Supplementary Tables 1- 4**

Supplementary Table 1: Gluconeogenic gene expression in livers of $WT^{fl/fl}$ or $FATA^{-/-}$ mice subjected to sham or HS.

Supplementary Table 2: Cytokine levels in $WT^{fl/fl}$ and $FATA^{-/-}$ mice subjected to sham or HS.

Supplementary Table 3: Cytokine levels in WT mice pretreated with vehicle or GS-9667 and subjected to sham or HS.

Supplementary Table 4: Cytokine and adipokine levels of *Aqp7*$^{-/-}$ mice subjected to sham or HS.

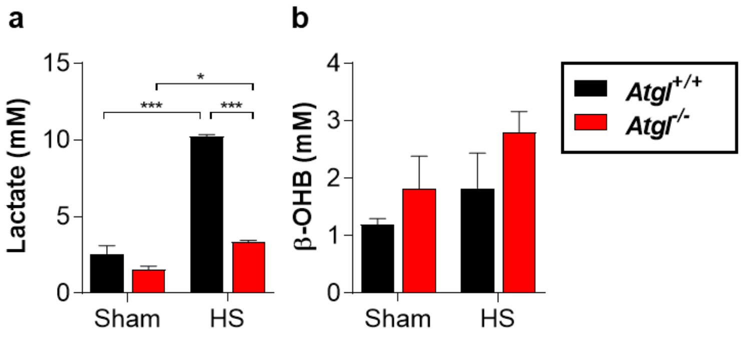

Supplementary Figure S1: Effect of genetic inhibition of global lipolysis on lactate and ketones. *Atgl*$^{+/+}$ and *Atgl*$^{-/-}$ mice were fasted 4 h, subjected to sham or HS, injected with saline or 0.5 U insulin in the inferior vena cava, and had tissues harvested after 4 min. (a) Serum lactate and (b) β-hydroxybutyrate (β-OHB) levels following sham or HS. $N = 3\text{-}4$. Data are shown as mean ± SEM. $^*P < 0.05$ and $^{***}P < 0.001$, as indicated. Statistical analyses were performed using two-way ANOVA. All comparisons differing by one variable were made and adjusted for with ANOVA, and all significant comparisons are denoted as such.

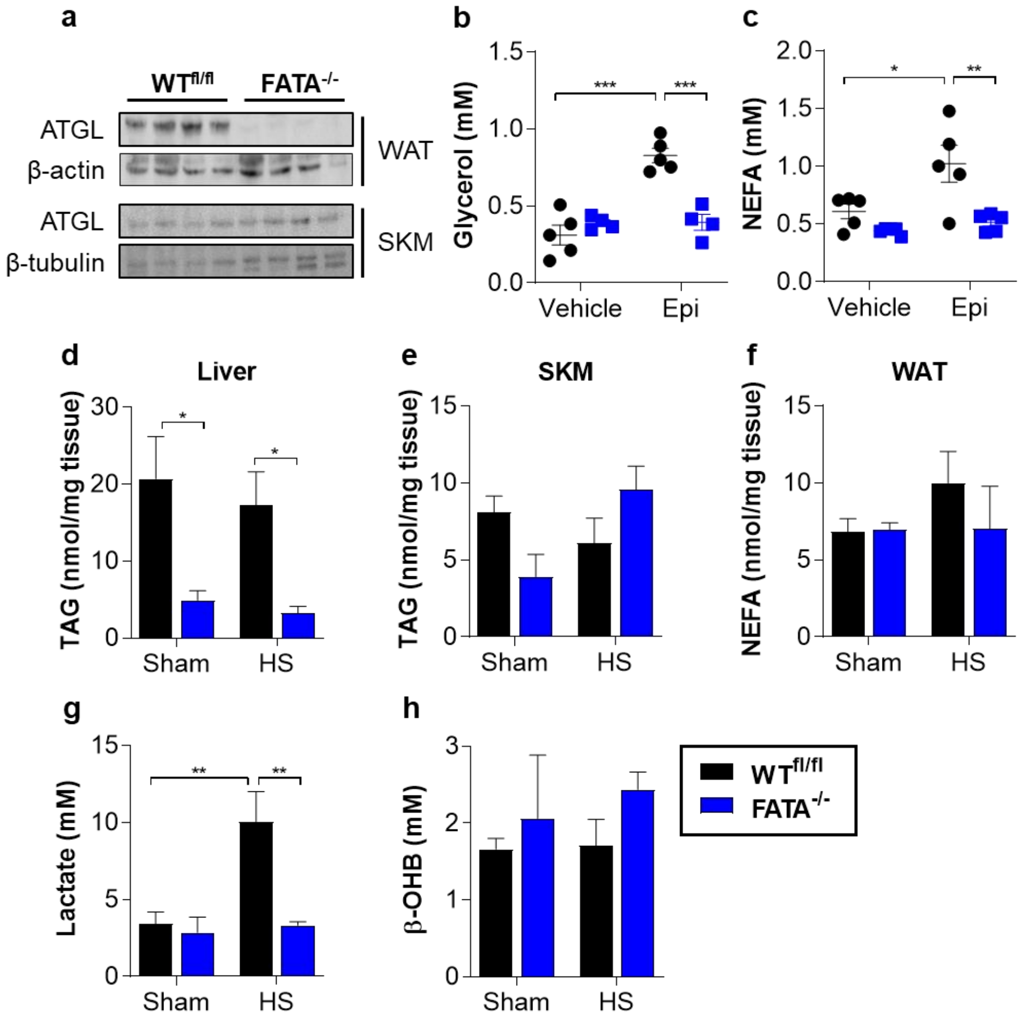

Supplementary Figure S2: Adipocyte-specific ATGL knockout and effect of genetic inhibition of adipocyte lipolysis on metabolite levels. (a) Immunoblots for white adipose tissue (WAT) and skeletal muscle (gastrocnemius, SKM) of WT$^{fl/fl}$ and FATA$^{-/-}$ mice demonstrating adipocyte-specific ATGL knockout. WT$^{fl/fl}$ and FATA$^{-/-}$ mice were fasted 4 h, injected i.p. with 2 mg/kg epinephrine, and had serum collected from the tail vein after 30 min. (b) Glycerol and (c) NEFA levels in serum after epinephrine injection. WT$^{fl/fl}$ and FATA$^{-/-}$ mice were fasted 4 h, subjected to sham or HS, injected with saline or 0.5 U insulin in the inferior vena cava, and had tissues harvested after 4 min. Tissue triglyceride levels in (d) liver and (e) skeletal muscle (gastrocnemius, SKM) following sham or HS. (f) NEFA levels in adipose tissue (WAT) following

sham or HS. (g) Serum lactate and (h) β-hydroxybutyrate (β-OHB) levels following sham or HS. $N = 3\text{-}7$. Data are shown as mean ± SEM. *$P < 0.05$, **$P < 0.01$, and ***$P < 0.001$ as indicated. Statistical analyses were performed using two-way ANOVA. All comparisons differing by one variable were made and adjusted for with ANOVA, and all significant comparisons are denoted as such.

a

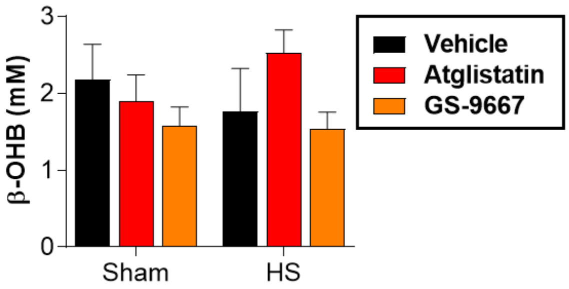

Supplementary Figure S3: Pharmacologic inhibition of lipolysis does not impact ketone production. C57BL6/J mice were fasted 4 h; pretreated either with vehicle, 1 mg/kg Atglistatin, or 5 mg/kg GS-9667; subjected to sham or HS; injected with saline or 0.5 U insulin in the inferior vena cava, and had tissues harvested after 4 min. (a) Serum β-hydroxybutyrate (β-OHB) levels following sham or HS. *N* = 4-5. Data are shown as mean ± SEM. Statistical analyses were performed using two-way ANOVA. All comparisons differing by one variable were made and adjusted for with ANOVA, and all significant comparisons are denoted as such.

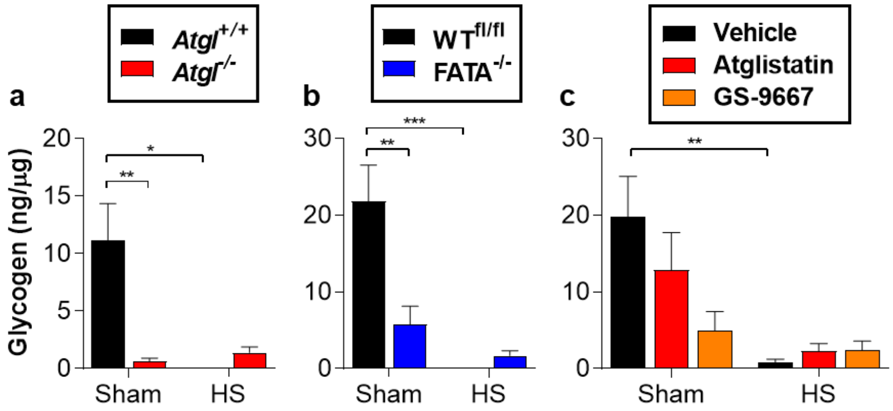

Figure S4: Glycogen concentrations following HS. Liver glycogen levels in (a) *Atgl*$^{+/+}$ and *Atgl*$^{-/-}$, (b) WT$^{fl/fl}$ and FATA$^{-/-}$, and (c) C57BL6/J mice pretreated either with vehicle, 1 mg/kg Atglistatin, or 5 mg/kg GS-9667 at 30 min after sham or HS. $N = 3$-8. Data are shown as mean ± SEM. *$P < 0.05$, **$P < 0.01$, and ***$P < 0.001$ as indicated. Statistical analyses were performed using two-way ANOVA. All comparisons differing by one variable were made and adjusted for with ANOVA, and all significant comparisons are denoted as such.

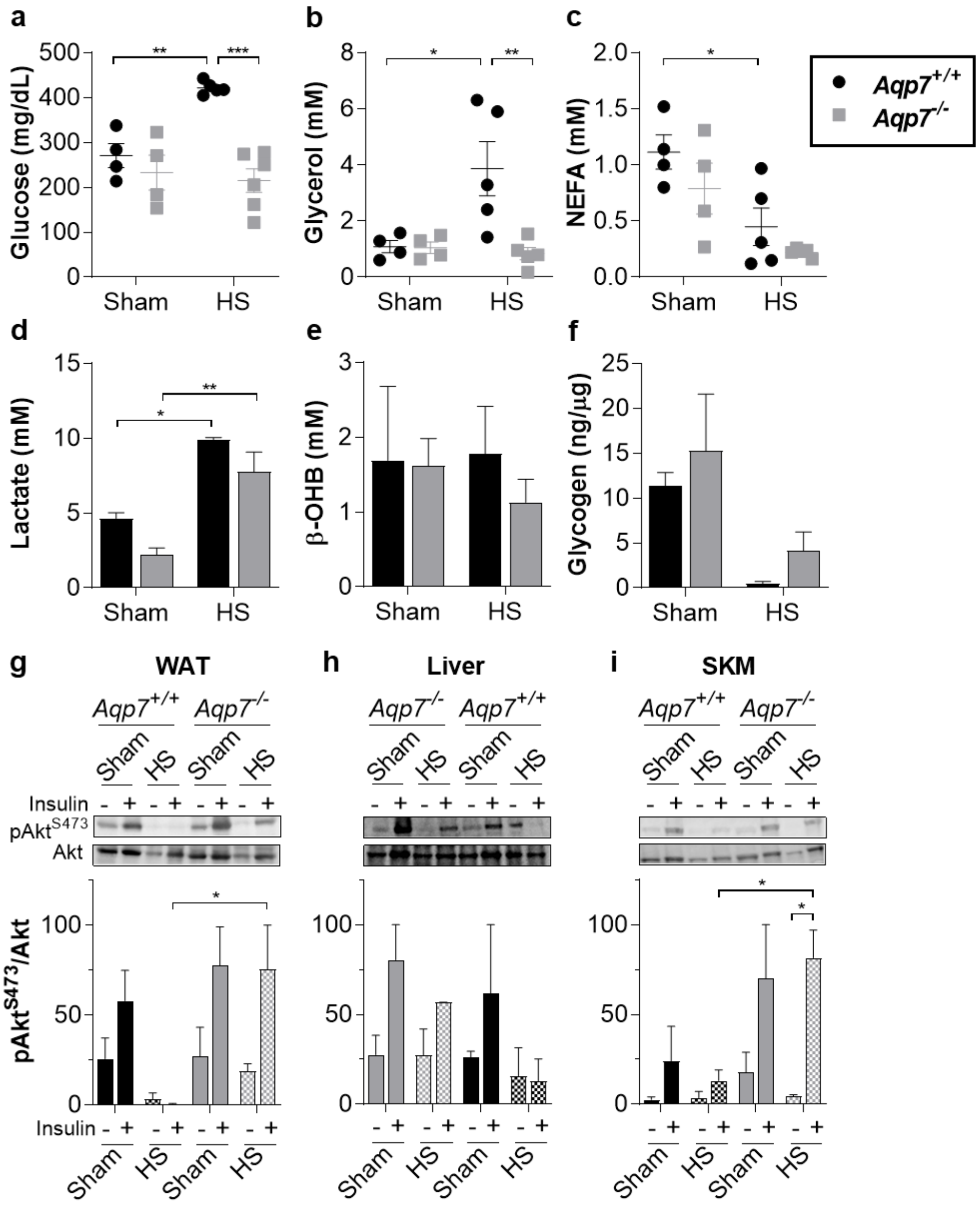

Supplementary Figure S5: Glycerol release during HS contributes to hyperglycemia. *Aqp7*$^{+/+}$ and *Aqp7*$^{-/-}$ mice fasted 4 h then subjected to sham or HS. After 30 min of sham or HS, *Aqp7*$^{+/+}$ and *Aqp7*$^{-/-}$ mice were injected with saline or 0.5 U insulin in the inferior vena cava, and tissues were harvested after 4 min. (a) Glucose, (b) glycerol, (c) NEFA, (d) lactate, and (e) β-hydroxybutyrate (β-OHB) levels in serum plus (f) liver glycogen levels following sham or HS.

Immunoblots and quantification (g) for white adipose tissue (WAT, epididymal), (h) liver, and (i) skeletal muscle (SKM, gastrocnemius) from *Aqp7*$^{+/+}$ and *Aqp7*$^{-/-}$ mice. Note that liver immunoblots were run in a different order than WAT and SKM and have been labeled as such. $N = 2\text{-}6$. Data are shown as mean ± SEM. $^{*}P < 0.05$, $^{**}P < 0.01$, and $^{***}P < 0.001$, as indicated. For a-f, statistical analyses were performed using two-way ANOVA. For g-i, image quantitations were normalized by setting largest value in each immunoblot to 100. Statistical analyses on relative values were performed using three-way ANOVA. All comparisons differing by one variable were made and adjusted for with ANOVA, and all significant comparisons are denoted as such.

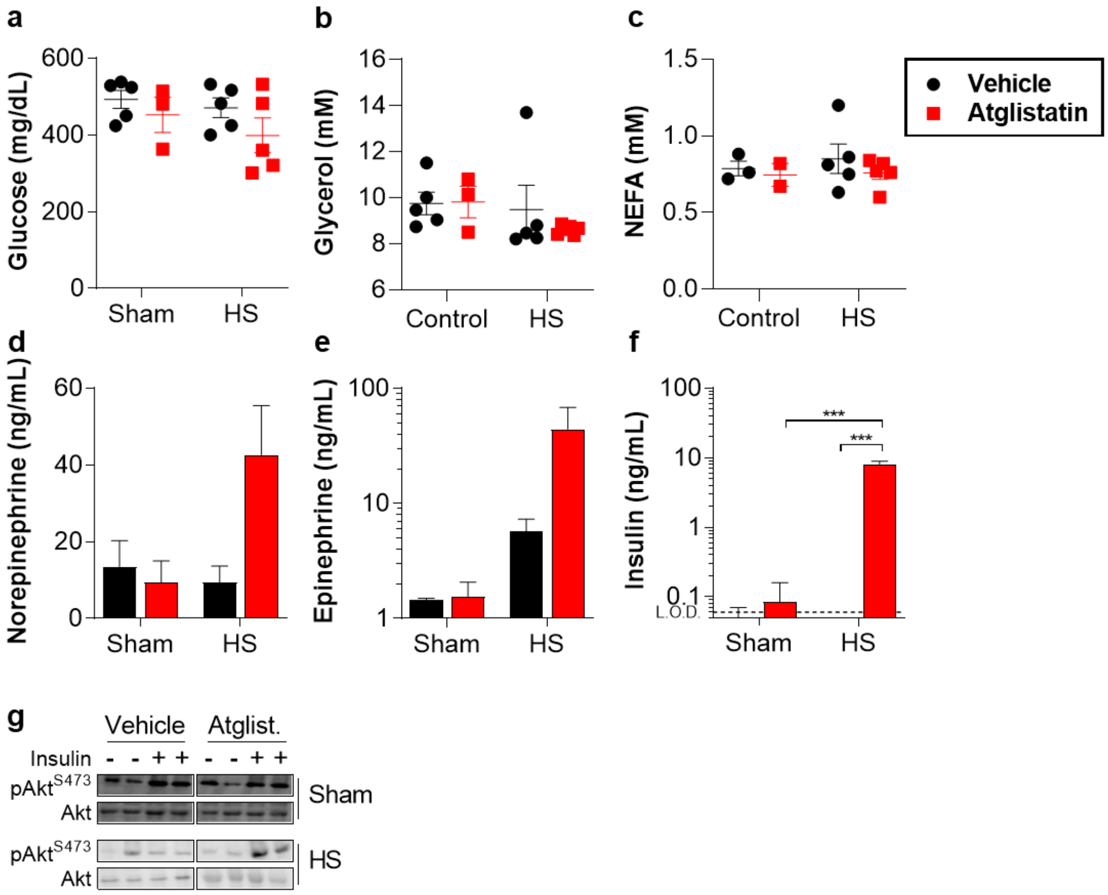

Figure S6: Glycerol infusion causes hyperglycemia in the absence of lipolysis. (a) Glucose, (b) glycerol, (c) NEFA, (d) norepinephrine, (e) epinephrine, and (f) insulin levels in serum of mice pretreated with vehicle or 1 mg/kg Atglistatin and an i.p. bolus of glycerol (3 mg/g) then subjected to sham or HS. After 30 min of sham or HS, the mice were injected with saline or 0.5 U insulin in the inferior vena cava and tissues harvested after 4 min. (g) Immunoblots for white adipose tissue. $N = 2\text{-}5$. Data are shown as mean ± SEM. ***$P < 0.001$. Statistical analyses were performed using two-way ANOVA. All comparisons differing by one variable were made and adjusted for with ANOVA, and all significant comparisons are denoted as such.

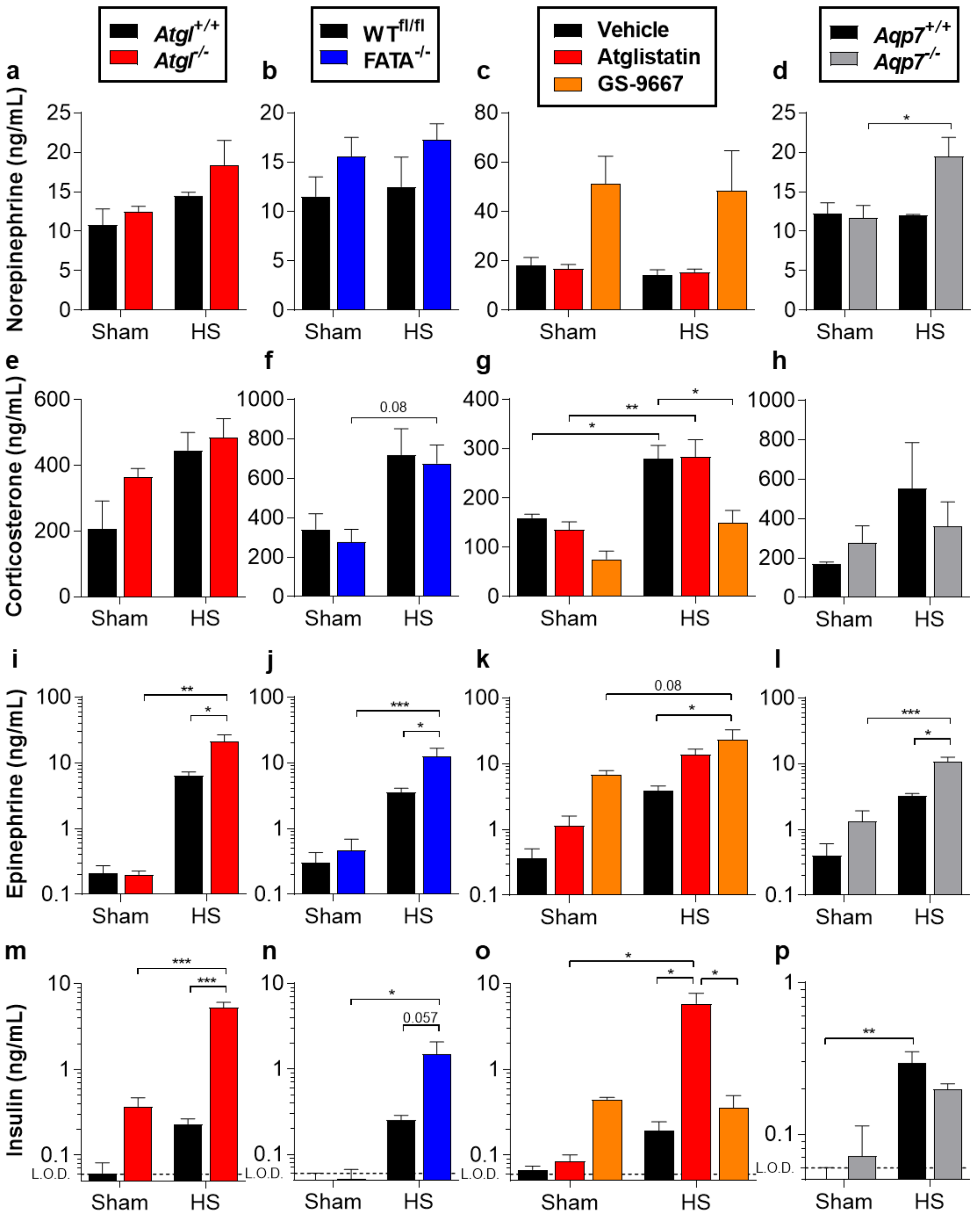

Supplementary Figure S7: Counterregulatory hormone and insulin levels during HS. Serum norepinephrine from (a) *Atgl*$^{+/+}$ and *Atgl*$^{-/-}$; (b) WT$^{fl/fl}$ and FATA$^{-/-}$; (c) C57BL6/J mice pretreated with vehicle, Atglistatin, or GS-9667; and (d) *Aqp7*$^{+/+}$ and *Aqp7*$^{-/-}$ mice subjected to sham or HS,

respectively. As in a-d, (e-h) corticosterone, (i-l) epinephrine, and (m-p) insulin. The limit of detection (LOD) for the insulin measurements is set at 0.05 ng/ml. $N = 2$-5. Data are shown as mean ± SEM. *$P < 0.05$, **$P < 0.01$, and ***$P < 0.001$, as indicated. Statistical analyses were performed using two-way ANOVA. All comparisons differing by one variable were made and adjusted for with ANOVA, and all significant comparisons are denoted as such.

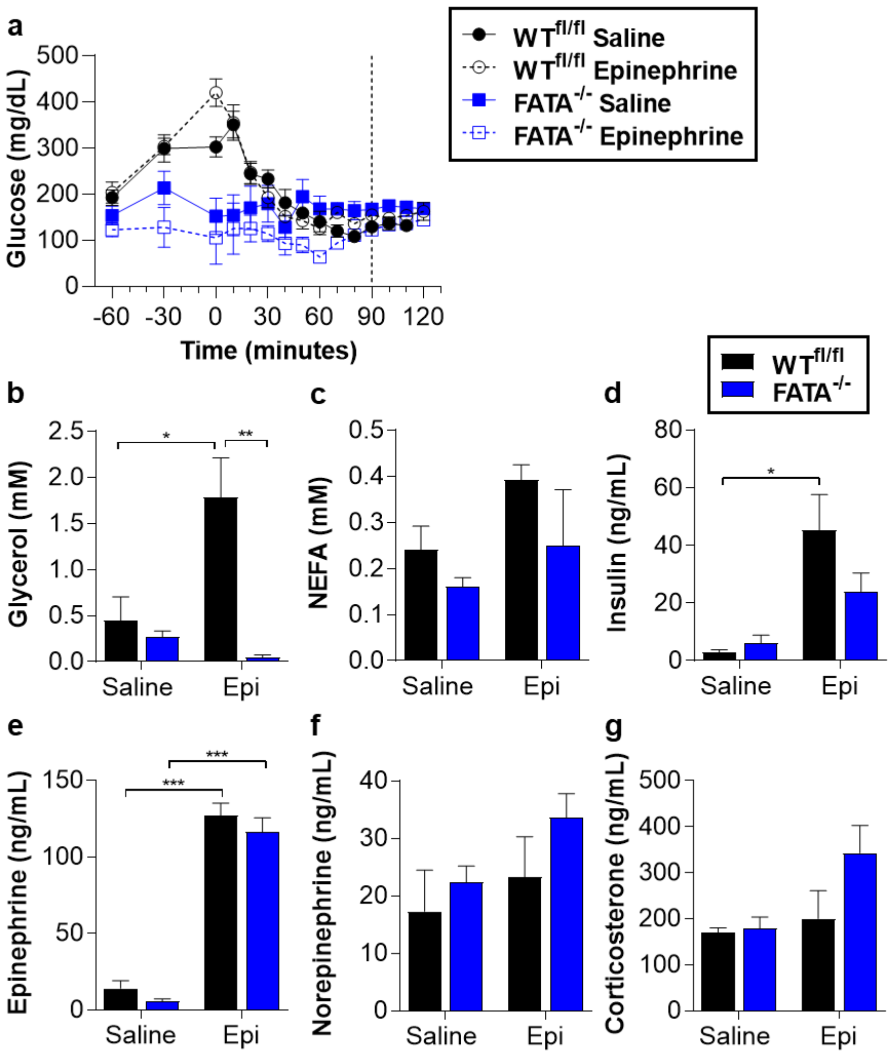

Figure S8: Glucose infusion rate, hormone, and lipolytic products in hyperinsulinemic-euglycemic clamps with epinephrine infusion in FATA$^{-/-}$ mice. WT$^{fl/fl}$ or FATA$^{-/-}$ mice fasted 2-4 h were infused with either saline or epinephrine (Epi, 1 µg/kg/min) followed by a hyperinsulinemic-euglycemic clamp (0-120 min). The mice then received a bolus of 2-deoxyglucose, and the clamp was continued for an additional 30 min (120-150 min). (a) Blood glucose levels during the course of the clamp. Dashed line indicates beginning of steady state (90-120 min). (b) Glycerol, (c) NEFA, (d) insulin, (e) epinephrine, (f) norepinephrine, and (g) corticosterone serum levels at the end of the clamp period (t = 150 min). *N* = 2-5. Data are shown

as mean ± SEM. *$P < 0.05$, *$P < 0.01$, and ***$P < 0.001$, as indicated. Statistical analyses were performed using two-way ANOVA. All comparisons differing by one variable were made and adjusted for with ANOVA, and all significant comparisons are denoted as such.

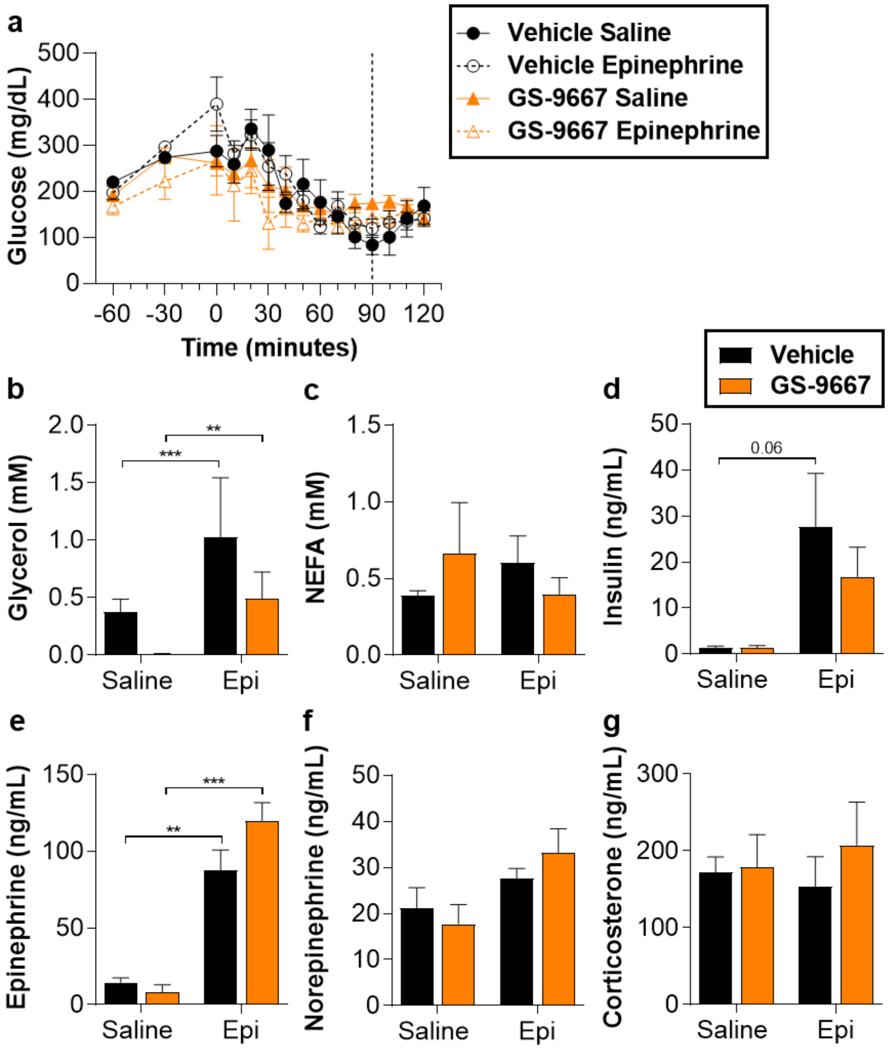

Figure S9: Glucose infusion rate, hormone, and lipolytic products in hyperinsulinemic-euglycemic clamps with epinephrine infusion after GS-9667 treatment. WT mice pretreated with vehicle or 5 mg/kg GS-9667 30 min prior to clamp were infused with either saline or epinephrine (Epi, 1 µg/kg/min) followed by a hyperinsulinemic-euglycemic clamp (0-120 min). The mice then received a bolus of 2-deoxyglucose, and the clamp was continued for an additional 30 min (120-150 min). (a) Blood glucose levels during the course of the clamp. Dashed line indicates beginning of steady state (90-120 min). (b) Glycerol, (c) NEFA, (d) insulin, (e) epinephrine, (f) norepinephrine, and (g) corticosterone serum levels at the end of the clamp period

(t = 150 min). *N* = 2-5. Data are shown as mean ± SEM. $**P < 0.01$ and $***P < 0.001$, as indicated. Statistical analyses were performed using two-way ANOVA. All comparisons differing by one variable were made and adjusted for with ANOVA, and all significant comparisons are denoted as such.

Supplementary Table 1:

	$WT^{fl/fl}$ Sham	$WT^{fl/fl}$ HS	$FATA^{-/-}$ Sham	$FATA^{-/-}$ HS
**PEPCK**	0.8 (0.1)	40.1 (4.9)	18.5 (7.0)	106.7 (19.1)*#$
**G6Pase**	0.4 (0.1)	55.9 (18.1)*	16.6 (6.3)	64.1 (10.5)*
**Glucokinase**	0.6 (0.1)	8.2 (1.5)*	0.7 (0.3)	1.6 (0.4)$
**PGC1-α**	0.5 (0.2)	5.0 (1.3)	9.2 (2.3)*	21.5 (3.0)*#$

*$P < 0.05$ compared to $WT^{fl/fl}$ Sham, #$P < 0.05$ compared to $FATA^{-/-}$ Sham, \$$P < 0.05$ compared to $WT^{fl/fl}$ HS

Supplementary Table 1: Gluconeogenic gene expression in livers of $WT^{fl/fl}$ or $FATA^{-/-}$ mice subjected to sham or HS. Phosphoenolpyruvate carboxykinase (PEPCK), glucose-6-phosphatase (G6Pase), glucokinase (GK), and peroxisome proliferator-activated receptor gamma co-activator 1-alpha (PGC-1α) gene expression are shown. Data expressed as relative gene expression compared to sham WT with SEM in parentheses. $N = 4$-6 per group. *$P < 0.05$ compared to $WT^{fl/fl}$ Sham, #$P < 0.05$ compared to $FATA^{-/-}$ Sham, \$$P < 0.05$ compared to $WT^{fl/fl}$ HS. Statistical analyses were performed using two-way ANOVA.

Supplementary Table 2:

Cytokine (pg/mL)	**WT$^{fl/fl}$ Sham**	**WT$^{fl/fl}$ HS**	**FATA$^{-/-}$ Sham**	**FATA$^{-/-}$ HS**
**IL-1α**	388.4 (117.3)	261.4 (38.2)	129.6 (8.1)	786.7 (158.3)
**IL-1β**	4.5 (1.4)	4.6 (0.9)	3.3 (1.4)	2.6 (0.7)
**IL-6**	4.5 (1.2)	60.5 (16.6)*	1.1 (0.5)	76.7 (30.9)#
**TNF-α**	1.8 (0.3)	11.4 (4.2)	2.2 (1.0)	2.3 (0.7)

*$P < 0.05$ compared to WT$^{fl/fl}$ Sham, #$P < 0.05$ compared to FATA$^{-/-}$ Sham.

Supplementary Table 2: Cytokine levels in WT$^{fl/fl}$ and FATA$^{-/-}$ mice subjected to sham or HS. Serum levels of IL-1α, IL-1β, IL-6, and TNF-α are shown. Data expressed as mean with SEM in parentheses. N = 4-6 per group. *$P < 0.05$ compared to WT$^{fl/fl}$ Sham, #$P < 0.05$ compared to FATA$^{-/-}$ Sham. Statistical analyses were performed using two-way ANOVA.

Supplementary Table 3:

Cytokine (pg/mL)	**Vehicle Sham**	**Vehicle HS**	**GS-9667 Sham**	**GS-9667 HS**
**IL-1α**	154.6 (107.3)	299.1 (198.0)	399.0 (40.6)	2101.3 (1629.9)
**IL-1β**	8.9 (6.5)	14.6 (8.2)	2.7 (0.4)	4.6 (1.3)
**IL-6**	5.6 (2.1)	25.3 (3.3)*	81.6 (20.2)	161.5 (42.5)
**TNF-α**	0.8 (0.02)	1.7 (0.5)	3.2 (1.4)	0.9 (0.1)

*$P < 0.05$ compared to $WT^{fl/fl}$ Sham.

Supplementary Table 3: Cytokine levels in WT mice pretreated with vehicle or GS-9667 and subjected to sham or HS. Serum levels of IL-1α, IL-1β, IL-6, and TNF-α are shown. Data expressed as mean with SEM in parentheses. $N = 3$ per group *$P < 0.05$ compared to Vehicle Sham. Statistical analyses were performed using two-way ANOVA.

Supplementary Table 4:

Cytokine (pg/mL)	***Aqp7***$^{-/-}$ **Sham**	***Aqp7***$^{-/-}$ **HS**	Adipokine (pg/mL)	***Aqp7***$^{-/-}$ **Sham**	***Aqp7***$^{-/-}$ **HS**
**IL-1α**	230.0 (131.3)	362.0 (91.0)	**Leptin**	861. 3(263)	202.3 (51)
**IL-1β**	12.2 (8.8)	5.6 (2.0)	**Adiponectin**	9.8 (1.7)	6.2 (0.6)
**IL-6**	2.8 (0.4)	16.6 (6.3)	**PAI-1**	3307 (618.1)	5784 (898.0)
**TNF-α**	4.0 (1.1)	1.5 (0.2)	**Resistin**$^{+}$	30.4 (3.38)	23.92 (9.16)

$^{+}$ Resistin expressed as ng/mL

Supplementary Table 4: Cytokine and adipokine levels of *Aqp7*$^{-/-}$ mice subjected to sham or HS. Serum levels IL-1α, IL-1β, IL-6, TNF-α, leptin, adiponectin, PAI-1, and resistin are shown. Data expressed as mean with SEM in parentheses. *N* = 3 per group

SUPPLEMENTARY DATA II

Full uncut gels

Adipocyte lipolysis drives acute stress-induced insulin resistance

Authors: Vidisha Raje[1], Katelyn W. Ahern[1], Brittany A. Martinez[1], Nancy L. Howell[2], Vici Oenarto[1,4], Mitchell E. Granade[1], Jae Woo Kim[1], Smanla Tundup[3], Katharina Bottermann[1], Axel Gödecke[4], Susanna R. Keller[2], Alexandra Kadl[1,3], Michelle L. Bland[1], Thurl E. Harris[1*]

Affiliations:

1. Department of Pharmacology, University of Virginia, Charlottesville, VA
2. Department of Medicine, Endocrinology and Metabolism, University of Virginia, Charlottesville, VA
3. Department of Medicine, Pulmonary and Critical Care Medicine, University of Virginia, Charlottesville, VA
4. Institute of Cardiovascular Physiology, Heinrich Heine University Düsseldorf, Germany

Fig. 1d (WAT)

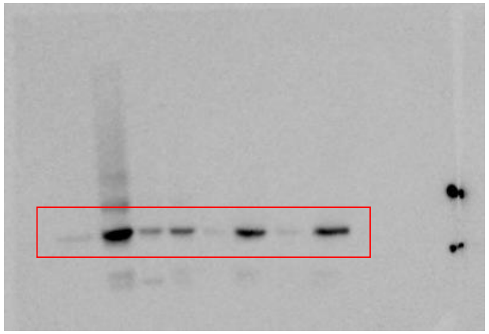

WT ATGL$^{-/-}$ pAkt^{S473}

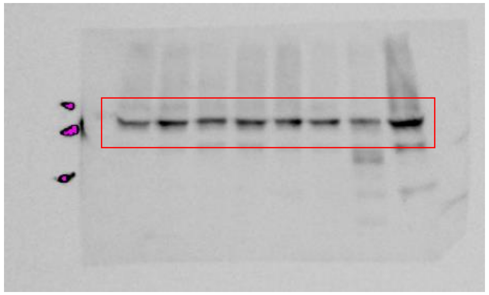

WT ATGL$^{-/-}$ Akt

Fig. 1e (Liver)

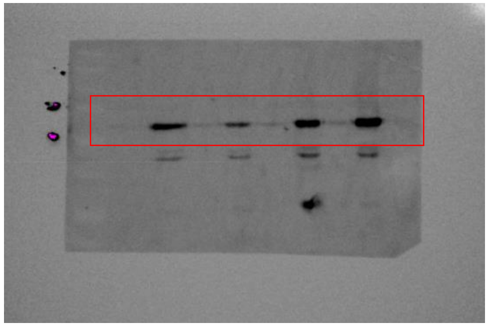

WT ATGL$^{-/-}$ pAkt^{S473}

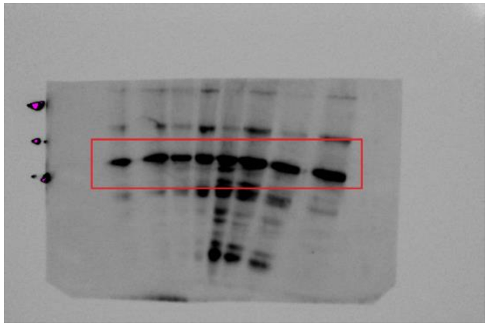

WT ATGL$^{-/-}$ Akt

Fig. 1f (SKM)

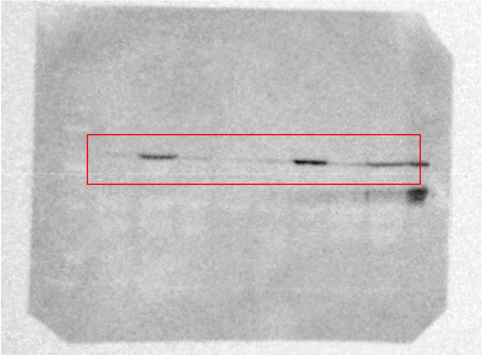

WT ATGL$^{-/-}$ pAkt^{S473}

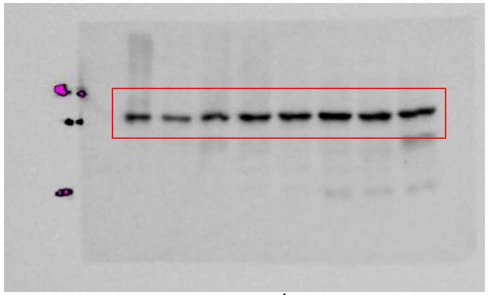

WT ATGL$^{-/-}$ Akt

Fig. 2d (WAT)

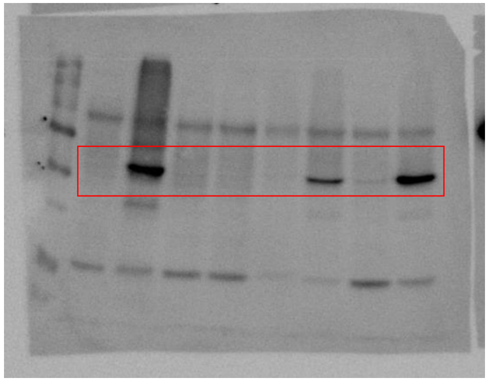

WT FATA$^{-/-}$ pAkt^{S473}

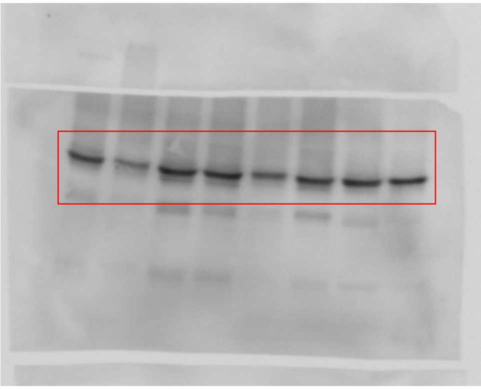

WT FATA$^{-/-}$ Akt

Fig. 2e (Liver)

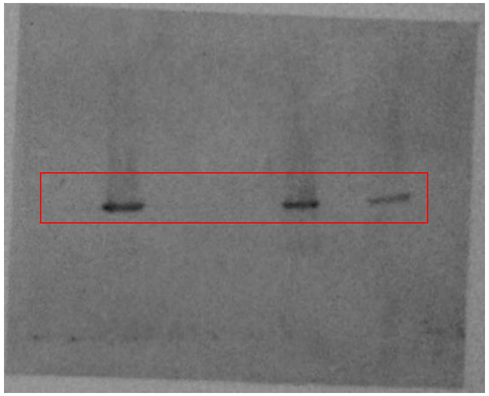

WT FATA$^{-/-}$ pAkt^{S473}

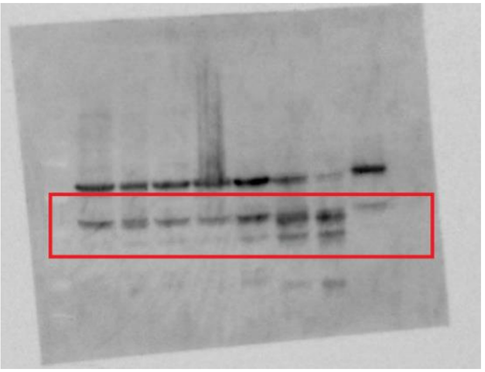

WT FATA$^{-/-}$ Akt

Fig. 2f (SKM)

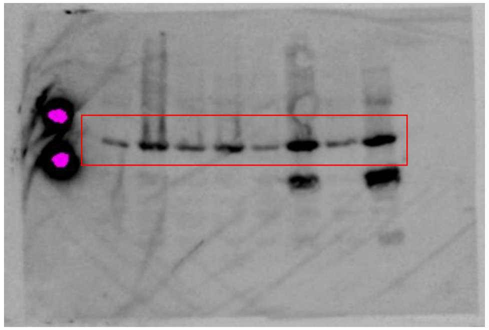

WT FATA$^{-/-}$ pAkt^{S473}

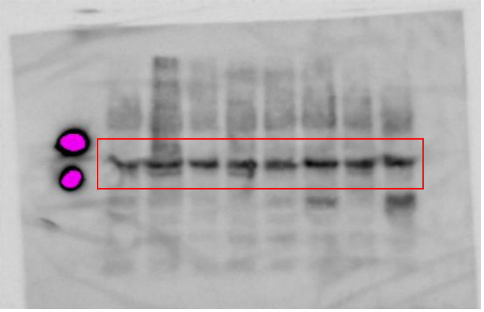

WT FATA$^{-/-}$ Akt

Fig. 3d (WAT)

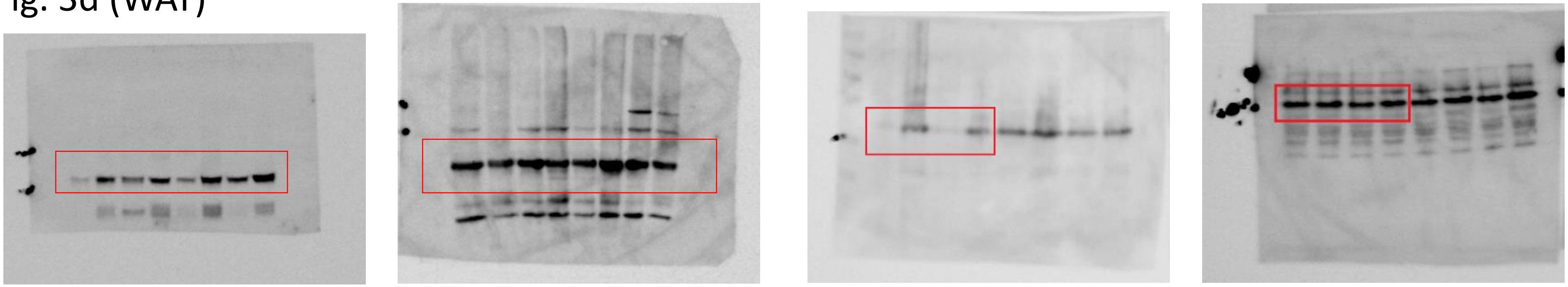

Vehicle Atglist pAkt^{S473} | Vehicle Atglist Akt | GS pAkt^{S473} | GS Akt

Fig. 3e (Liver)

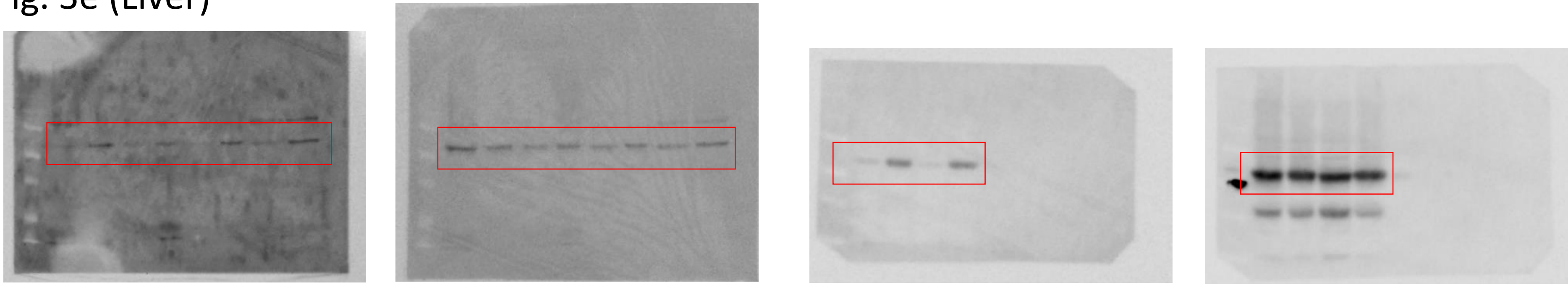

Vehicle Atglist pAkt^{S473} | Vehicle Atglist Akt | GS pAkt^{S473} | GS Akt

Fig. 3f (SKM)

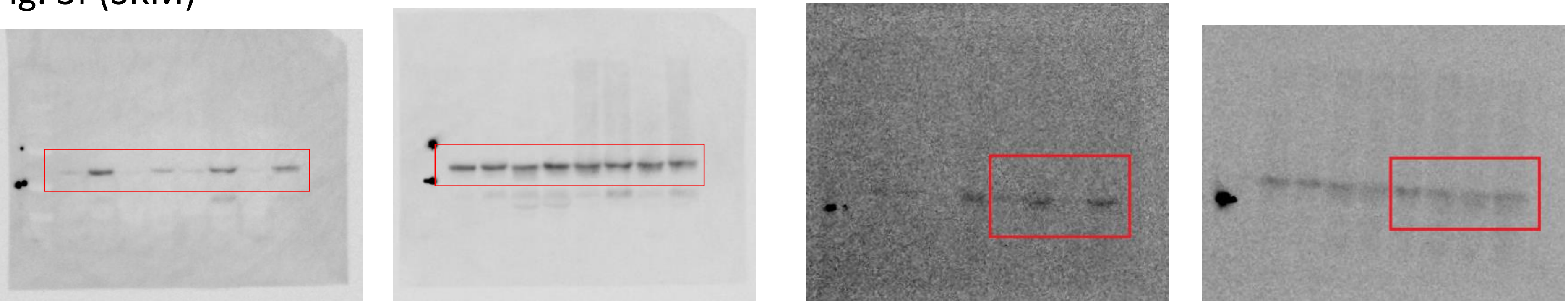

Vehicle Atglist pAkt^{S473} | Vehicle Atglist Akt | GS pAkt^{S473} | GS Akt

Fig. S2a

WAT

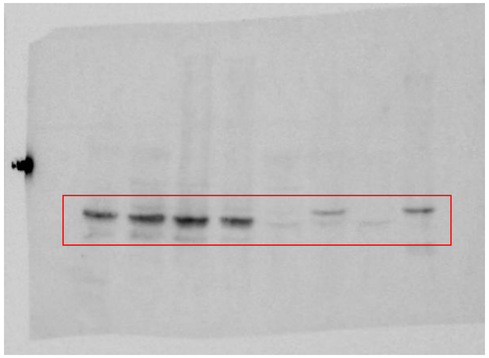

WT FATA$^{-/-}$ ATGL

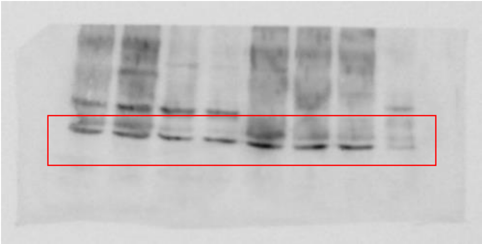

WT FATA$^{-/-}$ β-Actin

SKM

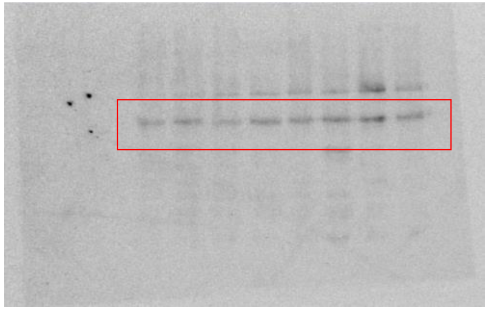

WT FATA$^{-/-}$ ATGL

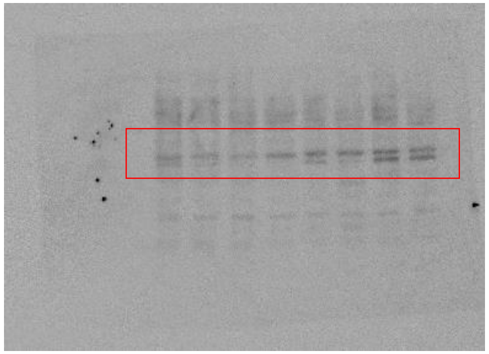

WT FATA$^{-/-}$ β-Tubulin

Fig. S5g (WAT)

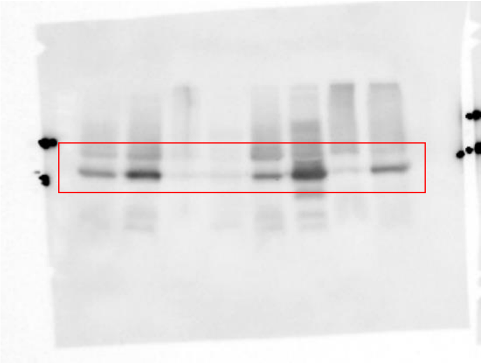

Aqp7$^{+/+}$ Aqp7$^{-/-}$ pAkt^{S473}

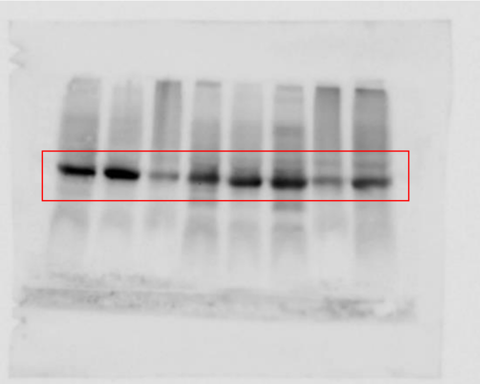

Aqp7$^{+/+}$ Aqp7$^{-/-}$ Akt

Fig. S5h (Liver)

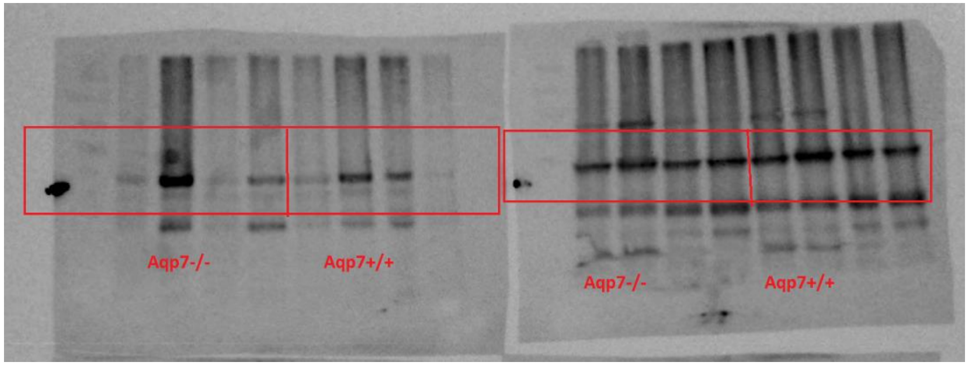

Aqp7$^{+/+}$ Aqp7$^{-/-}$ pAkt^{S473} Aqp7$^{+/+}$ Aqp7$^{-/-}$ Akt

Fig. S5i (SKM)

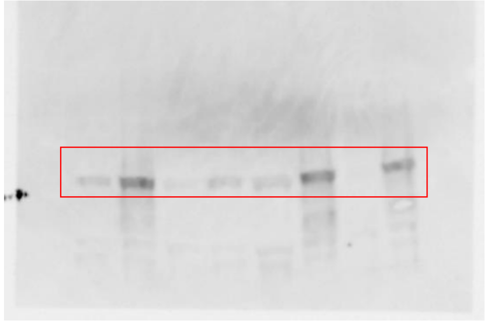

Aqp7$^{+/+}$ Aqp7$^{-/-}$ pAkt^{S473}

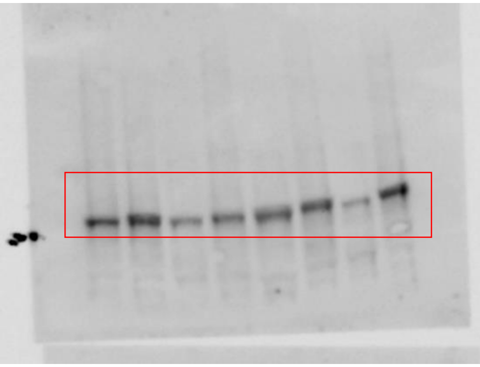

Aqp7$^{+/+}$ Aqp7$^{-/-}$ Akt

Fig. S6g

Sham

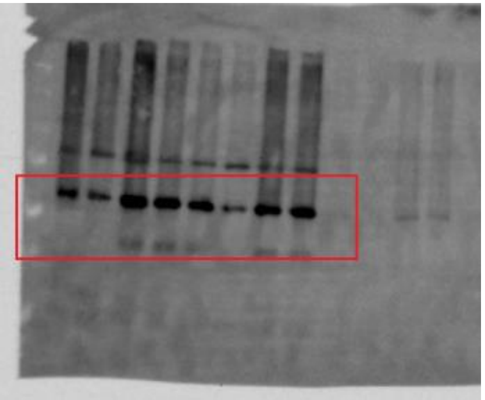

Vehicle Atglist pAkt^{S473}

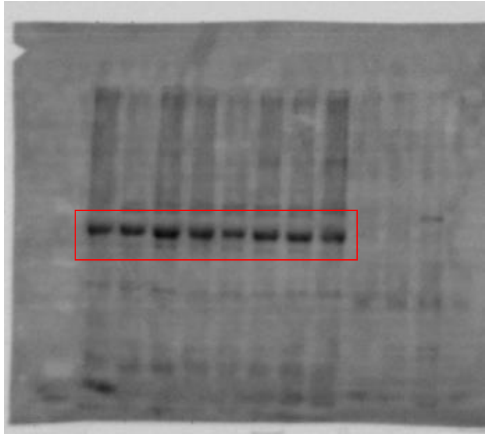

Vehicle Atglist Akt

HS

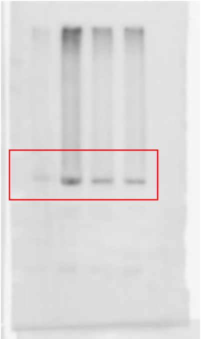

Vehicle pAkt^{S473}

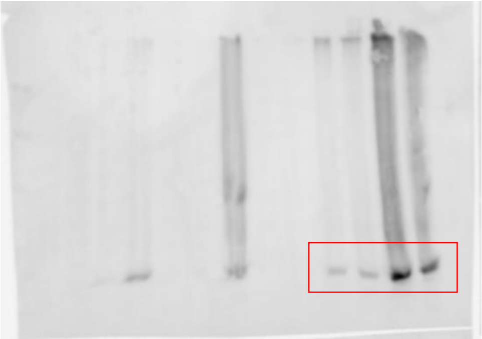

Atglist pAkt^{S473}

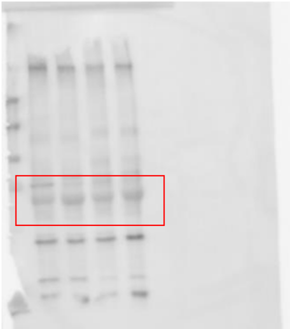

Vehicle Akt

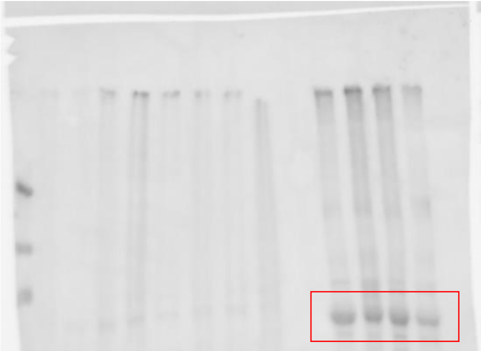

Atglist Akt
